# Three‐dimensional geometric morphometric analyses of humerus ecomorphology: New perspectives for paleohabitat reconstruction in carnivorans and ungulates

**DOI:** 10.1002/ar.25553

**Published:** 2024-08-09

**Authors:** Carmela Serio, Richard P. Brown, Marcus Clauss, Carlo Meloro

**Affiliations:** ^1^ Research Centre in Evolutionary Anthropology and Palaeoecology, School of Natural Sciences and Psychology Liverpool John Moores University Liverpool UK; ^2^ Clinic for Zoo Animals, Exotic Pets and Wildlife, Vetsuisse Faculty University of Zurich Zurich Switzerland

**Keywords:** discriminant function analysis, ecomorphology, humerus morphology, palaeohabitat reconstruction

## Abstract

Long bone ecomorphology has proven effective for paleohabitat reconstructions across a wide range of mammalian clades. Still, there is no comprehensive framework to allow interpretation of long bone morphological variation within and between different monophyletic groups. Here, we investigated the use of humerus morphometry to classify living members of the orders Carnivora and ungulates based on their preferred habitats. Using geometric morphometrics, we extracted three different kinds of humerus shape data describing interspecific variation with and without accounting for evolutionary allometry and phylogenetic signal. The traditional *a priori* categorization of species in open, mixed, and closed habitats was employed in combination with selected subsets of shape variables to identify the best‐predictive models for habitat adaptation. These were identified based on the statistical performance of phylogenetic and non‐phylogenetic discriminant analyses and then applied to predict habitats on a subsample of fossil species. Size‐free shape data combined with phylogenetic discriminant analyses showed the highest rate of accuracy in habitat classification for a combined sample of carnivorans and ungulates. Conversely, when the two groups were investigated separately, traditional shape data analyzed with phylogenetic discriminant function analyses provided models with the greatest predictive power. By combining carnivorans and ungulates within the same methodological framework we identified common adaptive features in closed habitat‐adapted species that show compressed epiphyses, while open habitat‐adapted species have expanded epiphyses. These morphologies evolved to allow significant degree of direction switches during locomotion in closed habitats compared to open habitat‐adapted species whose forelimb joints evolved to stabilize articulations for increasing speed.

## 1 INTRODUCTION

Ecomorphology focuses on the complex relationship between organismal morphology and function (Barr, 2018). Clarifying the nature of this relationship in living species is particularly relevant because it allows the interpretation of fossil morphologies and the inference of their mode of life with a quantitative approach. Ecomorphological studies have been effectively applied to a wide range of organisms such as fishes (Conith et al., 2020; Soria‐Barreto et al., 2019), lizards (Losos, 1990a, 1990b; Tinius et al., 2020), and mammals (see Elton et al., 2016 for a broad range of application in several clades).

The first ecomorphological studies were mainly focused on fossil bovids, abundant in paleoanthropological fossil sites, to infer the environmental context of human evolution (Barr, 2014; Kappelman, 1991; Kovarovic & Andrews, 2007; Plummer & Bishop, 1994; Scott et al., 1999). These works investigated postcranial morphology whose variation between living species should reflect locomotory behavior and habitat adaptations. Bovids from open habitats show extremely developed cursorial adaptations (elongation of distal elements in the limb bones), while species living in closed habitats exhibit a higher degree of joint mobility, likely to increase maneuverability (Croft et al., 2018; Etienne et al., 2021). Similar evidence has also been found in other limb elements such as the astragali (Barr, 2014, 2015; DeGusta & Vrba, 2003; Kovarovic & Andrews, 2007; Plummer et al., 2008, 2015), phalanges (DeGusta & Vrba, 2005; Louys et al., 2013), and metapodials (Plummer & Bishop, 1994; Scott et al., 1999).

In general, ungulates have a more specialized skeletal morphology compared to other mammalian groups, possibly as a result of optimized terrestrial locomotion (except for the semi‐aquatic specialists like the hippopotamus; Elton et al., 2016; Houssaye et al., 2021). Similar optimization for terrestrial locomotion is not apparent for their main predatory group: the Carnivora. Extant members of Carnivora exhibit a broad range of locomotory adaptations and behavior that is reflected in both the size and shape variation of long bones. Van Valkenburgh (1987) noted a series of traits, especially in the forelimb elements, which relate to scansorial and arboreal locomotion. Samuels et al. (2013) confirmed locomotion to be one of the primary drivers of variation in Carnivora limb proportionality. Harris and Steudel (1997) found prey capture adaptation to correlate highly with hind limb length suggesting the evolution of Carnivora limb length has been mostly influenced by selection for prey‐capture behavior. Also, the postcranial morphology of Carnivora has been interpreted in terms of habitat adaptation in living and fossil species (Figueirido et al., 2015; Lewis, 1997; Meloro, 2011; Meloro et al., 2013; Meloro & Louys, 2014; Polly, 2010; Van Valkenburgh, 1987).

The morphology of the limb bones changes quite broadly within mammals and each single bone has provided insights into how species adapt and interact with the external environment. Forelimbs are particularly relevant to predict habitat adaptations, with the humerus having been the focus of previous studies on suids, primates, bovids, and felids (Bishop, 1994; Elton, 2002; Kovarovic & Andrews, 2007; Meloro et al., 2013). Etienne et al. (2021) used geometric morphometrics to investigate long bone size and shape in bovids and showed that all elements of stylopodium and autopodium, except the tibia, are good predictors of habitat adaptation. Mallet et al. (2019) studied long bones in rhinoceroses and suggested that in this clade morphological variation is due mainly to changes in size. A similar level of understanding of the relationship between long bone size and shape and habitat type is currently lacking for the Carnivora, although linear measurements have been used to identify locomotor and climatic adaptations (Meachen et al., 2016; Samuels et al., 2013). Figueirido et al. (2015) demonstrated that Cenozoic climatic variations, which mainly produced changes in vegetation types, also impacted the evolution of elbow joint morphologies, affecting the hunting behavior of North American Canidae. Also, Meachen‐Samuels and Van Valkenburgh (2009) found that the humerus morphology is a good proxy to estimate preferred prey size in felids.

The humerus supports the anterior part of the body in quadrupedal mammals providing insertion for the muscles moving the forearms and manus. It articulates proximally with the scapula, hence impacting shoulder function, and distally with both the radius and ulna, giving information about the elbow joint flexion and extension ability. The shape and orientation of this bone vary among different mammalian groups, depending on their degree of forelimb mobility and stability that are indicative of habitat openness in both Carnivora and ungulates (Etienne et al., 2021; Janis & Figueirido, 2014; Martín‐Serra et al., 2016; Polly, 2007). Martín‐Serra et al. (2014) studying the forelimb morphologies in the Carnivora found that the greater tuberosity and humerus shaft curvature allow morphological inferences of the locomotor type and posture, respectively. In bovids, the robustness of the humerus predicts body mass, while the shape of the humerus head, tuberosities, trochlea, and epicondyles might change in relation to environmental adaptations (Etienne et al., 2021).

In this study we aim to test whether humerus shape is associated with habitat openness and whether it can be used as a proxy in paleovegetation reconstructions for fossil taxa. We focused on large [>7 kg, sensu Van Valkenburgh, 1987] species belonging to the most interactive mammalian groups: the ungulates (main group of prey) and the Carnivora (mainly predators) with the aim to understand if large predator–prey species living in the same environment share comparable humerus morphologies. We applied geometric morphometrics methods (GMM) to a combined sample of Carnivora and ungulates species and compared the performance of different shape data under multiple methodological scenarios when accounting for phylogenetic relationships and allometry.

Due to shared ancestry, interspecific datasets generally exhibit a strong phylogenetic signal in both phenotypic and ecological traits and this effect should be controlled for especially when testing predictive models (Barr & Scott, 2014; Harvey & Pagel, 1991; Revell, 2009). Barr (2014) identified two types of phylogenetic risk in ecomorphological studies. The ‘type A’ risk represents the chance that living species differ ecologically from their fossil relatives, which can be difficult to mitigate against. The ‘type B’ risk represents the chance that similar morphologies might be the result of shared ancestry and not an environmental adaptation. This latter risk can be avoided by measuring and controlling for the degree of phylogenetic signal in the ecomorphological traits under study. Equally important to the phylogenetic signal can also be the size‐related variation in shape data due to biomechanical constraints imposed by gravity on the skeletal system (Etienne et al., 2021; Mallet et al., 2019; Martín‐Serra et al., 2014).

By assembling a database of humerus shape variables belonging to Carnivora, Artiodactyla, and Perissodactyla our investigation covered different subsets of data to maximize separation between living species due to habitat preference. Additionally, we hypothesized that, because Carnivora and ungulates living in closed habitats share reduced constraints on forelimb movements relative to species living in open habitats (Etienne et al., 2021; Janis & Figueirido, 2014; Martín‐Serra et al., 2016), their humerus morphology can be informative for paleohabitat reconstruction. Our expectation was that habitat preferences based on a combined sample of Carnivora and ungulates will be comparable with reconstructions based on clade‐specific datasets. We equally explored several combinations of shape data for the humerus coupled with the traditional *a priori* categorization of living species into open, mixed, and closed habitats (Janis & Wilhelm, 1993; Kappelman, 1988; Meloro, 2011). These combinations of different shape data and habitat categories were analyzed with phylogenetic and non‐phylogenetic functional discriminant models. These methods were already shown to be useful in developing accurate predictive models which extended to fossil species using reliable habitat categorizations (Barr, 2018; Gruwier & Kovarovic, 2022, 2023; Kovarovic et al., 2011, 2021; Kovarovic & Andrews, 2007). Motani and Schmitz (2011), implemented the discriminant analyses allowing to model the method accounting for the phylogenetic information. To select the best phylogenetic and non‐phylogenetic model, we implemented the method proposed by Kovarovic et al. (2011) that identifies the best predictive model based on living species. These models are finally applied to reconstruct habitat preferences in a selection of fossil taxa belonging to the orders Carnivora, Artiodactyla, and Perissodactyla.

### 1.1 Fossil specimens and localities

For this work we investigated 36 specimens of undeformed fossil humeri representing 29 species of Carnivora, Artiodactyla, and Perissodactyla. These remains are merely representative of major Holarctic paleontological sites (*n* = 21) from several regions including Spain, France, Poland, Romania, Italy, Greece, Germany, California, and Florida. Our sampling was mainly restricted due to the status of completeness, preservation, and availability from the visited museum institutions (see below and Appendix A).

The Carnivora humeri belonging to *Canis dirus* and *Smilodon fatalis* came from the Rancho la Brea deposits (California, USA). Even if the two species inhabited the same environment, *C. dirus* has been predicted to prefer open habitat, while *S. fatalis* possibly preferred to hunt prey from closed habitats (DeSantis et al., 2019). The humerus belonging to *Megantereon cultridens* was excavated at Saint‐Vtallier, Drôme (France), while the humeri of *Ursus spelaeus* came from three different localities (Igric‐barlang, Romania; Aitzkirri, Spain; Jerzmanowice Cave Alkurz, Poland). These two species were predicted to prefer closed and open habitats, respectively (Christiansen & Adolfssen, 2007; Meloro, 2011; Meloro & de Oliveira, 2019). The *Pseudaelurus* sp. humerus was found in Le Grive Saint Alban (Isère, France). The paleoenvironmental reconstruction of this site is difficult due to the mixed faunal assemblage discovered in its deposits, but the ungulates assemblage supported an open environment (Aiglstorfer et al., 2023). However, *Pseudaelurus* sp. was capable of climbing (Domingo et al., 2017), and it is equally possible that it preferred closed environments. Controversial is also the habitat preference of *Homotherium* sp. The morphological characters of this extinct sabertooth cat suggest it was adapted to open habitats (DeSantis et al., 2021; Meloro, 2011), however, Antón et al. (2005) concluded it may have preferred mixed environments. Among Carnivora specimens, we also studied humeri belonging to *Cephalogale geoffroyi* (Allier, France), *Metatomarctus canavus* (Thomas Farm, Florida), and *Cynelos* cf. *lemanensis* (Allier, France). These samples are particularly interesting because reconstructions of habitat adaptations have yet to be attempted for them.

The Perissodactyla fossil data include the humeri of five rhinocerotids and two equid species. Two specimens, belonging to *Brachypotherium aurelianense*, came from Ronville (Loiret, France). According to the body size of the genus, this species was indirectly predicted to inhabit closed environments (Antoine, 2002; Guérin, 1980; Heissig, 1989). The humeri of *Diceratherium asphaltense* (Saulcet, Allier) and *Dihoplus megarhinus* (Sables de Montpellier) came also from two French localities. A study on the fossil rhinoceros found in the deposits of Saulcet suggests that *D. asphaltense* was adapted to live in open forested areas close to bodies of water (Becker, 2009). By contrast, we did not find any paleohabitat inferences for the rhinoceros from Sables de Montpellier (*D. megarhinus*). Yet, the woolly rhinoceros (*Coelodonta antiquitatis*) was known to inhabit open steppe (Stefaniak et al., 2021). The fossil equids humeri are from *Equus stenonis* and *Hipparion* sp. *E. stenonis* humerus was found in the Senèze (France) site, and according to Cirilli et al. (2021) equids from the French localities inhabited mixed environments with both open and closed areas. The *Hipparion* sp. humeri are also controversial because they were excavated at the site of Batallones‐10 where animals of different habitat preferences were recovered altogether (Martin‐Perea et al., 2021).

A study on the dental enamel of equids found at Layna, the paleontological site where the bovid *Gazella borbonica* was found in Spain, suggested the paleoenvironment was characterized by open habitat (Domingo et al., 2009). Another Spanish site, Los Valles de Fuentidueña, where the humerus belonging to the giraffid *Decennatherium pachecoi* was found, was inferred to be characterized by open habitat due to its faunal assemblage (Aiglstorfer et al., 2023). Among the other Artiodactyla humeri, the specimens of *Eucladoceros senezensis*, *Metacervocerus rhenanus*, and *Gallogoral meneghini* came from the French site of Senezè. Paleoenvironmental reconstruction of Ceyssaguet, another site where *E. senezensis* was recovered suggested this species was adapted to closed environment (Kaiser & Croitor, 2004). The same inference has been done for *G. meneghini* (Bellucci & Sardella, 2015; Eastham et al., 2016; Strani et al., 2015). The humeri belonging to the fossil deer *Euprox furcatus* and *Micromeryx flourensianus* were found in the German site of Steinheim. These two specimens showed controversial habitat reconstruction because the small *E. furcatus* was predicted to prefer mixed habitats (Aiglstorfer et al., 2014), while the tusked deer *M. flourensianus* closed (Aiglstorfer et al., 2014; Eastham et al., 2016). The morphology of *Candiacervus cretensis*, the humerus of which was found in Rethymnon (Kreta, Greece), suggested this species was open‐adapted (Caloi & Palombo, 1996; de Vos, 2000). Our dataset also includes three species of large bovids. Both the humeri belonging to *Leptobos etruscus* were collected at the site of Le Strette (Val D'Arno, Italy) while the humerus belonging to *Bos primigenius* was collected in Val di Chiana (Italy). We did not find paleoenvironmental reconstruction for these Italian sites, however, paleohabitat inferences on *L. etruscus* suggested this was an open‐adapted species (Bocherens et al., 2015; Strani et al., 2018). Yet, analyses of the aurochs (*B. primigenius*) tooth microwear showed this bovid was capable of feeding on leaves and trees, suggesting it might have inhabited marginal habitats of forested areas (Mead et al., 2014; Schulz & KaiSer, 2007). The two humeri belonging to *Bison priscus*, housed in the Hungarian Natural History Museum, came from unknown localities. However, according to the inference made by Bocherens et al. (2015), this species preferred open habitats. Unknown localities are also for the extinct moose *Alces brevirostris*, the ancient moschid *Pomelomeryx gracilis*, and the South American camelid *Lama castelnaudi*. For these species, we could not find paleohabitat reconstructions. All the paleohabitat inference found in literature were summarized in Table 1.

**TABLE 1:** Summary of paleohabitat reconstruction for a selected number of fossil large mammal based on the literature.

Species	Existing paleohabitat inference	Reference
*Alces brevirostris*	‐	
*Bison priscus*	Open	Bocherens et al. (2015)
*Bos primigenius*	Closed	Mead et al. (2014); Schulz and KaiSer (2007)
*Brachypotherium aurelianense*	Closed	Antoine (2002); Guérin (1980); Heissig (1989)
*Candiacervus cretensis*	Open	Caloi & Palombo (1996); de Vos (2000)
*Canis dirus*	Open	DeSantis et al. (2019)
*Cephalogale geoffroyi*	‐	
*Coelodonta antiquitatis*	Open	Stefaniak et al. (2021)
*Cynelos* cf. *lemanensis*	‐	
*Decennatherium pachecoi*	Open	Aiglstorfer et al. (2023)
*Diceratherium asphaltense*	Open	Becker (2009)
*Dihoplus megarhinus*	‐	
*Equus stenonis*	Mixed	Cirilli et al. (2021)
*Eucladoceros senezensis*	Closed	Kaiser and Croitor (2004)
*Euprox furcatus*	Mixed	Aiglstorfer et al. (2014)
*Gallogoral meneghini*	Closed	Bellucci and Sardella (2015); Eastham et al. (2016); Strani et al. (2015)
*Gazella borbonica*	Open	Domingo et al. (2009)
*Hipparion*	Mixed	Martin‐Perea et al. (2021)
*Homotherium*	Open/Mixed	DeSantis et al. (2021); Meloro (2011)/(Antón et al. (2005)
*Lama castelnaudi*	‐	
*Leptobos etruscus*	Open	Bocherens et al. (2015); Strani et al. (2018)
*Megantereon cultridens*	Closed	Christiansen and Adolfssen (2007); Meloro (2011)
*Metacervocerus rhenanus*	Closed	Kaiser and Croitor (2004)
*Metatomarctus canavus*	‐	
*Micromeryx flourensianus*	Closed	Aiglstorfer et al. (2014); Eastham et al. (2016)
*Pomelomeryx gracilis*	‐	
*Pseudaelurus*	Open/Closed	Aiglstorfer et al. (2023)/Domingo et al. (2017)
*Smilodon fatalis*	Closed	DeSantis et al. (2019)
*Ursus spelaeus*	Open	Meloro and de Oliveira (2019)

## 2 MATERIALS AND METHODS

### 2.1 Data collection

We collected 192 humeri specimens from adult, non‐pathological individuals belonging to 124 species (*n* = 41 Carnivora, *n* = 65 Artiodactyla, *n* = 18 Perissodactyla) of which 95 were extant and 29 extinct. Only 187 were identified at species level, while three *Hipparion* specimens (all coming from the same paleontological site), one *Homotherium*, and one *Psedaelurus* were identified only at genus level.

Sampled specimens (Appendix A) were from the following institutions: the Manchester Museum (MM, Manchester, UK), National Museums of Scotland (NMS, Edinburgh, UK), Liverpool World Museum (LWM, Liverpool, UK), Museo Nacional de Ciencias Naturales (MNCN, Madrid, Spain), Hungarian Natural History Museum (HNHM, Budapest, Hungary), and Naturhistorisches Museum Basel (NMB, Basel, Switzerland).

Each specimen was photographed approximately 200 times in dorsal, lateral, and ventral views using a digital SLR (Nikon D3300, lens 18–55 mm). Most images were taken at a 1‐m distance with a fixed focal length of 55 mm. Agisoft Metashape software was employed to build the humeri 3D models using photogrammetry. Once created, the models were rescaled with Meshlab software using maximum bone length as a proxy to scale the 3D model along the *x*, *y*, and *z* axes (Falkingham, 2011). The models have a mean resolution of 472,036 triangles and 236,413 vertices.

In addition, we included 46% of 3D sample from online databases (www.morphosource.org and www.sketchfab.com). Although these models were built under different resolutions and with different instrumentation (e.g., surface laser scanners, CT‐scans), previous sensitivity analyses based on small and medium‐sized mammalian skulls demonstrated that this introduces only a marginal error when extracting morphological data at an interspecific scale (see Giacomini et al., 2019; Marcy et al., 2018). To account for species shared evolutionary history in the analyses, an informal supertree was assembled from different sources (Álvarez‐Carretero et al., 2021; Cerdeño, 1996; Nyakatura & Bininda‐Emonds, 2012; Zrzavý et al., 2018; Zurano et al., 2019). The assembled tree included all 124 species studied (Appendix B).

Habitat type has been shown to impact interspecific morphological variation of the postcranial skeleton in both carnivorans and ungulates (Elton et al., 2016; Etienne et al., 2021; Kovarovic et al., 2021; Kovarovic & Andrews, 2007; Kovarovic & Scott, 2014; Meloro et al., 2013). The broad habitat classification includes open, mixed, and closed categories based on the openness of the environment. We retrieved data to assign habitat categories for living species from the IUCN Red List of Threatened Species (https://www.iucnredlist.org/) and Animal Diversity Web (https://animaldiversity.org/) (Appendix C). Because both databases often contained different habitat descriptions for some species, we needed to interpret the information available in order to assign them in one of our three openness categories. For example, Animal Diversity Web includes desert, dune, savanna, and grassland as the preferred environment for the cheetah (*Acinonyx jubatus*). Equally the IUCN Red List of Threatened Species in the habitat description of the cheetah reports: “*Cheetah are found in a wide range of habitats and ecoregions, ranging from dry forest and thick scrub through to grassland and hyperarid deserts*.” Combing this information, we considered *A. jubatus* to prefer open habitat types.

### 2.2 3D geometric morphometrics method (3D GMM)

**FIGURE 1:**
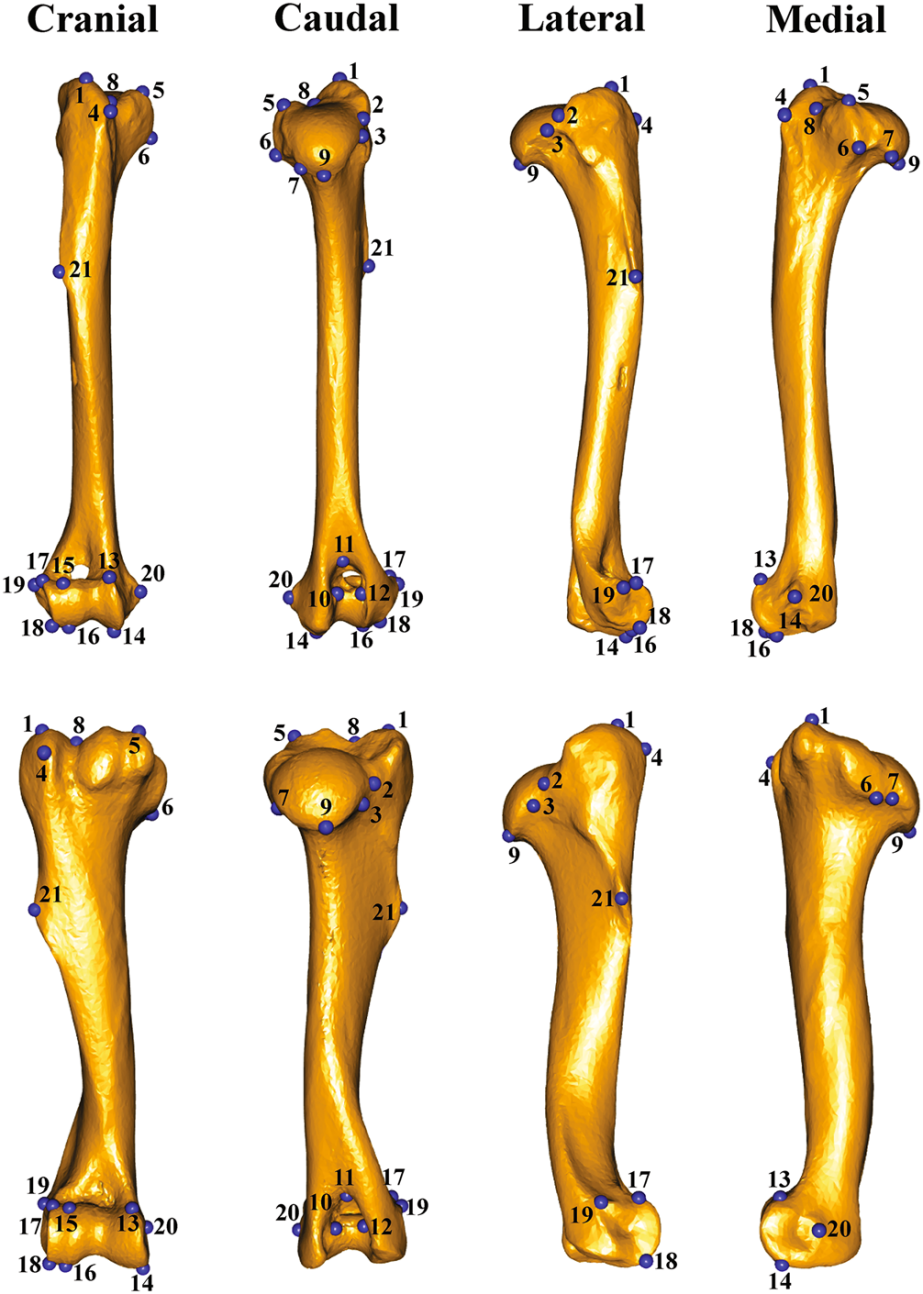
Landmark configuration applied to the humerus of *Canis lupus* (upper), representative of Carnivora sample, and to the humerus of *Lama guanicoe* (bottom), the representative of the ungulates sample. The humerus 3D models were built using photogrammetry. Blue dots represent anatomical landmarks in cranial, caudal, lateral, and medial views.

A set of 21 homologous landmarks were digitized on each humerus 3D model using the software Landmark (v. 3.0, IDAV 2002–2005). The landmark configuration was chosen to capture different morphological characters of carnivorans and ungulates and was based on the previous works of Martín‐Serra et al. (2016), Michaud et al. (2020), and Etienne et al. (2021). It followed the anatomical characters described in Table 2 and Figure 1.

**TABLE 2:** Landmarks description.

1	Most dorsal point of the greater tubercle
2	Point of junction between the greater tubercle and the humeral head
3	Most laterocaudal point of contact between the greater tuberosity and the humeral head
4	Most cranial point of the greater tubercle
5	Most dorsal point of the lesser tubercle
6	Most ventral point of the lesser tubercle
7	Most mediocaudal point of contact between the lesser tuberosity and humeral head
8	Maximum concavity of the intertubercular groove
9	Ventrocaudal tip of the humeral head
10	Most medial point of the olecranon fossa
11	The medial tip point of the olecranon fossa
12	The most lateral point of the olecranon fossa
13	The most lateral and proximal point of the trochlea
14	The most lateral and distal point of the trochlea
15	The most proximal contact point between the trochlea and the capitulum
16	The most distal contact point between the trochlea and the capitulum
17	The most lateral and proximal point of the capitulum
18	The most lateral and distal point of the capitulum
19	The most lateral tip of the lateral epicondyle
20	The most lateral tip of the medial epicondyle
21	Tip of the deltopectoral crest

Generalized Procrustes Analysis (GPA) was used to remove the effects of spatial orientation and size from the 3D landmark configurations, via translation, rotation and scaling of the original landmark coordinates (Rohlf & Slice, 1990). Because our sample primarily consisted of the right humerus, when a left humerus was needed to be studied, the collected landmarks were reflected along the *x*‐axis prior to any further analyses. For nine species (indicated in Appendix A), we estimated missing landmarks using the function *fixLMtps* (Schlager, 2017). The GPA was carried out using the *gpagen* function in the R package geomorph (Adams et al., 2016). Centroid size (CS) was obtained from the raw landmark coordinates as a proxy for specimen size and defined as the square root of the sum of the squared distances from each landmark to the centroid of each configuration (Zelditch et al., 2012). Because we collected from one (*n* = 57 species) up to five specimens per species, both shape and size data were averaged by species in case multiple specimens were available for the same taxon (*n* = 67).

To reduce the data dimensionality, simplify the interpretability, and store the maximum amount of information from the new set of shape coordinates we applied the function *gm.prcomp* (R package geomorph; Adams et al., 2016) which computes two different kinds of Principal Component Analysis (PCA).

1. Traditional PCA (PCA): this method decomposes the shape information into new orthogonal axes of maximum variation called Principal Components (PC). Here, the PC returned from the PCA were abbreviated as tPCs.
2. Phylogenetic PCA (phylo‐PCA) *sensu* Collyer and Adams (2020): this method takes into account the non‐independence of data due to species phylogenetic relationships when constructing axes of greatest variance. The variance–covariance matrix was centered via generalized least squares (GLS) and the GLS residuals were transformed using the phylogenetic transformation matrix to completely remove the phylogenetic signal (Collyer & Adams, 2020). The new phylogenetic PCs here were abbreviated as phylo‐PCs.

To account for allometry, we used the function *procD.lm* in the geomorph package (Adams et al., 2016). This function computes the Procrustes ANOVA to quantify how much shape variation was imputable to the size variation and assess the significance of the model. We repeated this analysis accounting for the phylogenetic relationship between species using the function *procD.pgls* (Adams et al., 2016). In this case, data were assumed to evolve under the Brownian Motion model and a matrix extracted from the phylogenetic tree was used to transform both response and independent variables. To account for allometric variation, we computed size‐free shape data by regressing shape coordinates versus naturally log‐transformed CS (lnCS). Residuals were submitted to the traditional PCA, in order to obtain size‐free PCs which we abbreviate as sfPCs. Procustes ANOVA was also employed to investigate if size differ among the different habitat types.

The phylogenetic signal was computed for each set of shape coordinates applying the function *physignal* in the geomorph R package (Adams et al., 2016). This function implements Blomberg et al. (2003)'s kappa (*K*) statistic with multivariate datasets (*K*‐mult; Adams, 2014). K estimates the degree of phylogenetic signal according to what is expected under the Brownian Motion model of evolution. Blomberg *K* equal or close to 1 means closely related species show the greatest similarity according to what is expected under the Brownian Motion model. Blomberg *K* lower/higher than 1 means closely related species are more similar/dissimilar between them than would be expected under Brownian Motion (Blomberg et al., 2003; Elton et al., 2016). This function also returns the levels of significance based on the permutation test. To compute the phylogenetic signal of the habitat category, we quantified Pagel's lambda [a parameter that varies between 0.0 = no signal, and 1.0 = maximum phylogenetic signal] using the function *fitDiscrete* embedded in the geiger R package (Harmon et al., 2008). All the analyses mentioned in this section and hereafter were carried out for the entire sample and separately for Carnivora and ungulates, and Procrustes superimposition was performed independently for every subsampled dataset.

### 2.3 Predict habitat preferences for fossil species

**FIGURE 2:**
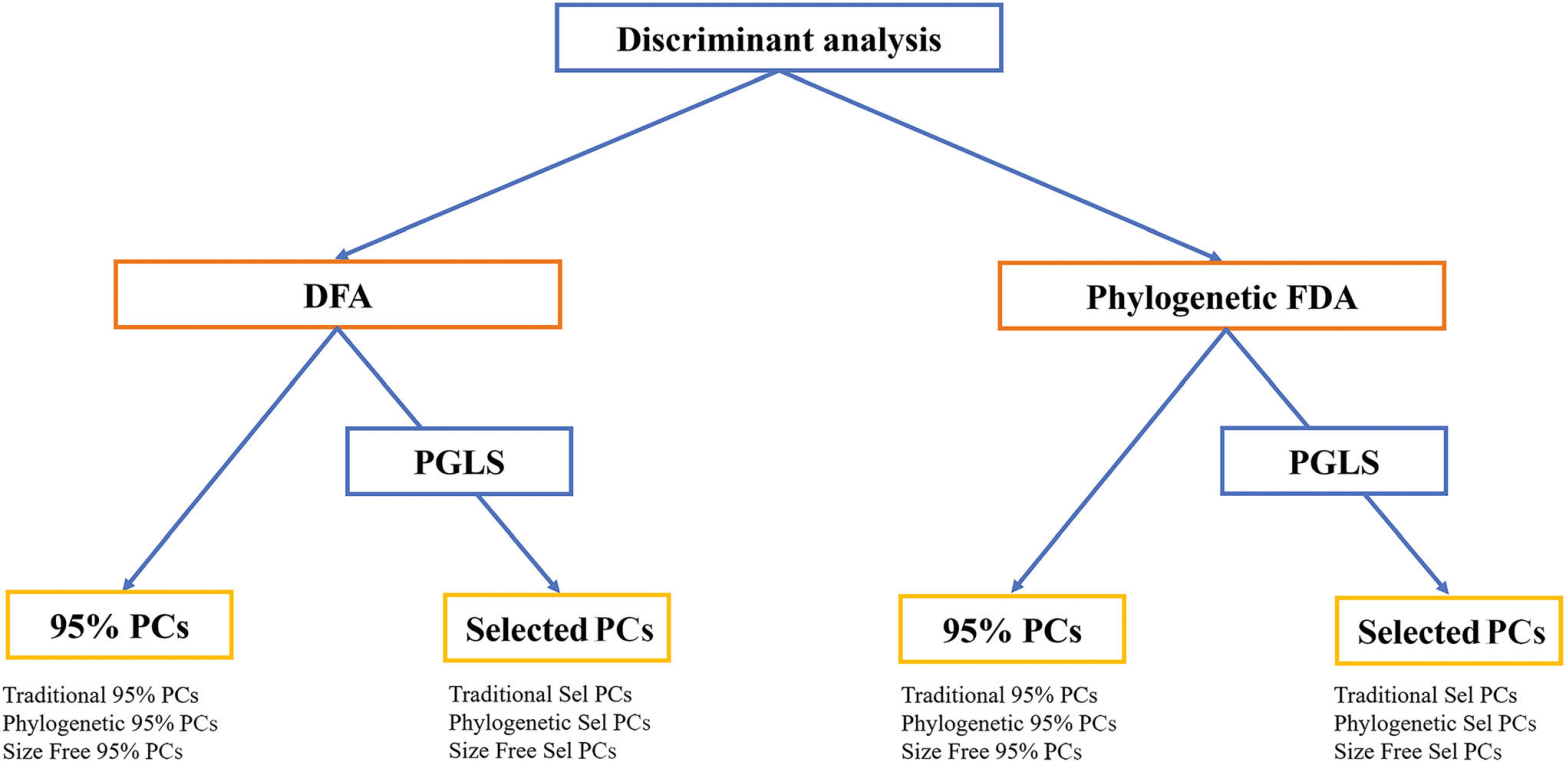
Analytical workflow of the discriminant functional analyses applied to the total sample (number of extant species = 94) and the selected subsamples of Carnivora (number of extant species = 32) and ungulates (number of extant species = 63). The selected models were then applied to infer habitat openness for the fossil species of the whole sample (*n* = 29) and Carnivora (*n* = 9) and ungulates (*n* = 20) subsamples.

To predict habitat preference in fossil species, we employed discriminant function analysis (DFA) which takes predictors (i.e., the PC vectors obtained from shape variables) and returns new axes which maximize the separation of predefined groups of individuals, which in this case corresponded to habitat categories (Figure 2).

We performed both phylogenetic and non‐phylogenetic informed DFA twice, first using a subset of PCs explaining 95% (i.e., 95% tPCs, 95% phylo‐PCs, and 95% sfPCs) of the cumulative variance of the shape, then using subsets of PCs selected among all the tPCs, phylo‐PCs, and sfPCs separately that described significant relationships between humerus shape of living species and habitat categories (Barr, 2015). To identify the subset of selected PCs, we employed the Phylogenetic Generalized Least Squares (PGLS) regression which takes into account the non‐independence of phenotypic data between species resulting from shared evolutionary history.

The habitat category to be used in the PGLS model was transformed in a dummy variable. The new variable is a matrix formed by *n* rows as the number of observations, and three columns one for each habitat category. For each category (i.e., open, closed, and mixed) the variable shows 1 for the presence of the species in the category and zero for the absence of the species in the category. Then the dummy matrix was standardized, using the R function *scale*, to a mean of 0 and standard deviation of 1. Each dummy variable was regressed against each PC one at a time. PGLS regressions were computed in R using the function *pgls* in the caper R package (Orme et al., 2018) that allows quantification of the phylogenetic signal (lambda) in the model residuals by relaxing the Brownian Motion assumption (Revell, 2010).

Both 95% PCs and selected PCs, belonging to the different sets of shape variables (i.e., tPCs, phylo‐PCs, and sfPCs), were used as independent variables to predict habitat group membership in fossil species using DFA (Kovarovic et al., 2011). DFA was computed by the *lda* function in the MASS R package (Venables & Ripley, 2013) and *phylo.fda* function (Motani & Schmitz, 2011) was equally employed to account for the phylogenetic effect. *phylo.fda* function takes into account a lambda value estimated with the function *optLambda* (Motani & Schmitz, 2011). The input data of this function are the predictor variables (in our case, shape data represented the different type of PC vector scores), habitat category, and the phylogenetic tree of living species. Internally, *optLambda* creates a vector of 100 lambda values spanning from 0 to 1. Each value of lambda is used to correct the phylogenetic bias of both categorical (*Y*) and shape (*X*) variables. For each couple of phylogenetically corrected *X* and *Y*, the goodness of fit is evaluated by computing the residual sum of squares (RSS; Martins & Hansen, 1997). The function returns the value of lambda, used to correct the traits, which returned the lowest RSS. This lambda represents the optimal value for the correlation between habitat categories (*Y*) and shape (*X*). It may vary between 0 (absence of phylogenetic bias) and 1 (presence of phylogenetic bias; Motani & Schmitz, 2011). Lambda was used to remove the phylogenetic bias from both shape variables and the ecological category into the *phylo.fda* function.

The *phylo.fda* function computes the Flexible Discriminant Analysis (FDA) which was implemented by adjusting for the phylogenetic signal lambda. FDA works by transforming the response variable (i.e., the categorical variable) using the linear regression so that ecological groups are separated along a regression line. Therefore a simple DFA is applied to maximize group separation (Hastie et al., 1994). PGLS was used instead of linear regression in the FDA to transform the response variable.

In both DFA and phylo‐FDA, the rate of correct classification cases (i.e., the hit rate) was determined using cross‐validation. However, the classification rate alone cannot be compared across different DFAs/phylo‐FDAs because different predictors are likely to affect the results. Because this work aimed to evaluate which set of shape variables was the best to infer habitat preferences in fossil species, we implemented a new R function called *compare.dfa* (Appendix D) to assess the goodness of fit for both DFA and phylo‐FDA. The *compare.dfa* function is based on the procedure described in Kovarovic et al. (2011) and compares the hit rates and TAUs obtained while reducing the number of predictors two at a time. The hit rate is the percentage of correctly classified observations after cross‐validation, while TAU is a correction applied to the hit rate. TAU considers the probability of correctly classifying cases by chance and is generally lower than the hit rate. The resulting discriminant model outputs from our data were compared with DFA outcomes from simulated data. During the simulation, group affiliation was randomly reassigned to erase differences between groups and the analyses were repeated for all predictors and their subsets 100 times. The average and 95 percentiles of both correct classification rates and TAUs were computed for the 100 simulations. According to the protocol of Kovarovic et al. (2011), the function generates two plots showing changes in hit rates and TAUs, respectively. The hit rates and TAUs are plotted versus the number of predictors used in the analysis. The average and 95 percentile confidence intervals are equally represented. An additional plot also compares the similarity of real and simulated groups for each set of simulations. When the black line (representing the real data) in the plot is close or internal to the range of simulated data (the gray lines), it indicates that there are no differences between the real and simulated data, and the model performance is weak. When *compare.dfa* is used to compare phylo‐FDAs, it also produces a plot describing the change in the lambda parameter.

*compare.dfa* returns a table including the hit rates and TAUs for all the discriminant analyses computed (i.e., using all the predictors and reduced) while reducing the number of predictors two at the time and the percentage of similarity between the simulated habitat category and the observed. This result is returned for the real and the simulated data. In addition, we implemented the Kovarovic et al. (2011) procedure to assess the goodness of fit for each DFA model. *compare.dfa* generates two *p*‐values that refer to the number of times the real average hit rate and TAU are higher (or lower) than the averaged hit rate and TAU of the simulated dataset, divided by the number of simulations. The hit rate and TAU are significantly higher than the simulated metrics, when the *p*‐value is higher than 0.975. Because a good DFA model must be above the upper range of simulated data, the function calculates the mean differences between the distribution of the real data and the upper range of simulation. When the mean difference is larger than 0, the real data exceeds the simulated range. When the mean difference is smaller than 0, the real values are within or below the 95% confidence interval. We computed the analyses for all kinds of shape variables (i.e., as retrieved from both traditional and phylogenetic PCA and size‐free residuals).

Among the significant models, the best one for each data sample was chosen based on both the hit rate and TAU parameters, as well as the mean differences. Once selected, the best model was used to infer habitat adaptations in fossil species. In the case of DFA, predictions were made using the *predict.lda* function MASS R package (Venables & Ripley, 2013) while for phylo‐FDA the function *phylo.fda.pred* was used (Motani & Schmitz, 2011). *phylo.fda.pred* takes as predictors the shape variables and phylogenetic tree of the whole sample (i.e., living *plus* fossil species), and the observed habitat category group (i.e., representing only living species). It essentially corrects the phylogenetic bias of the input data in the same way as *phylo.fda*, but does not take the phylogeny into account when it makes fossil predictions. Finally, we used the function *shape.predictor* (geomorph R package; Adams et al., 2016) to restore visualization of the humerus shape along the discriminant axes.

## 3 RESULTS

### 3.1 Geometric morphometrics

**FIGURE 3:**
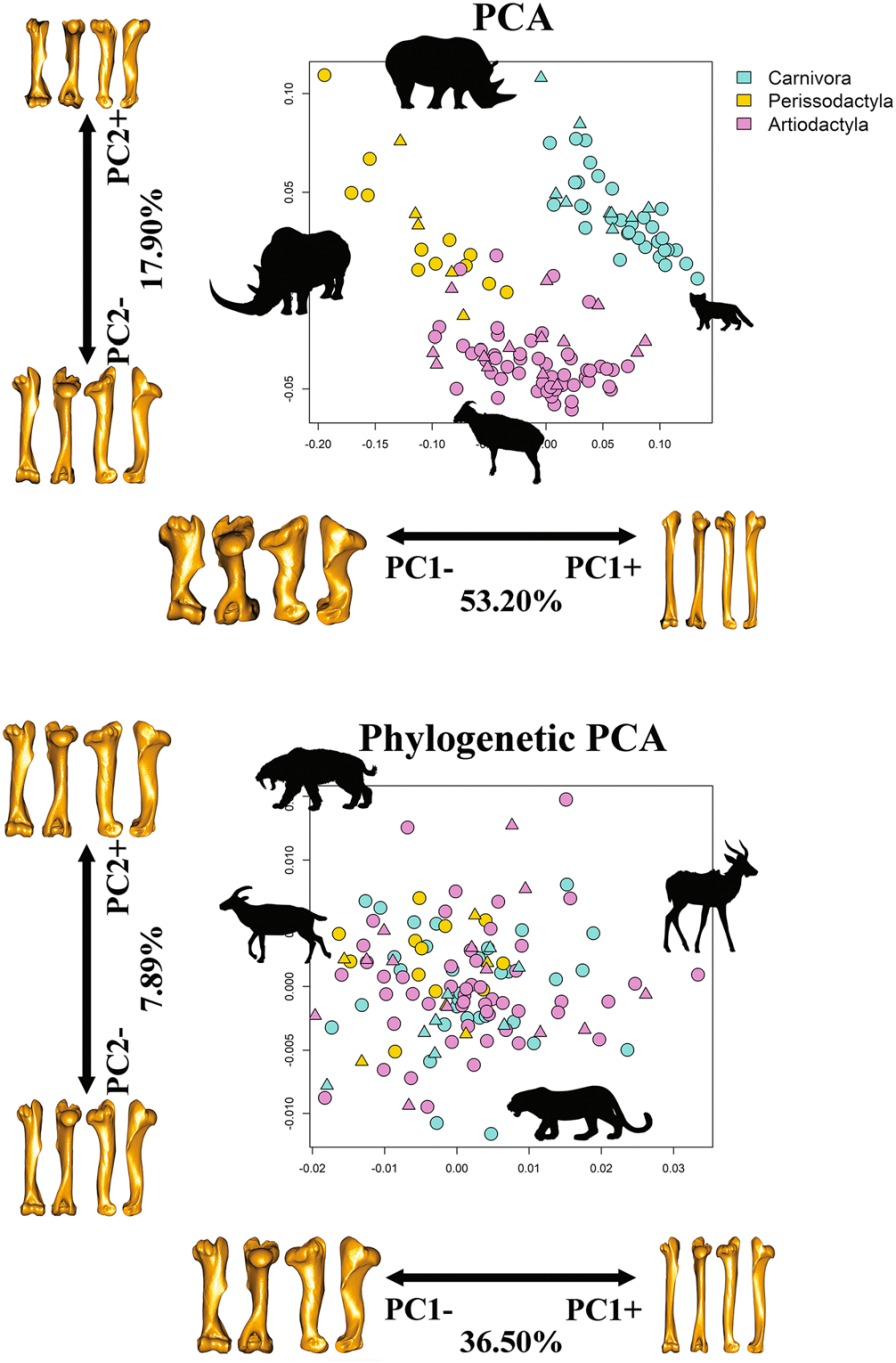
Scatterplot of humerus shape variation along PC1 and PC2 axes. Species are color coded according to order. Deformation warping of humerus 3D models along the extreme PC scores was generated using as reference specimen *Lama guanicoe*. Upper: PC1 versus PC2 scatter plot based on traditional PCA. The *Coelodonta antiquitatis* silhouette relates to the most extreme negative tPC1 score, *Felis margarita* silhouette is on the extreme positive tPC1 score, *Hemitragus jemlahicus* silhouette associates with extreme negative score of tPC2 while *Ceratotherium simum* is on the extreme positive tPC2. Lower: PC1 versus PC2 scatterplot based on the phylogenetic PCA. *Gallogoral meneghini* silhoutte is on the extreme negative phylo‐PC1 while *Tragelaphus spekii* on the extreme positive phylo‐PC1, *Panthera pardus* silhouette is associated with extreme negative phylo‐PC2 and *Smilodon fatalis* on the extreme positive phylo‐PC2 score. Deformation warping of humerus 3D models are shown in cranial, caudal, lateral, and medial views. Extant species are represented by circles and extinct species by triangles.

Both tPCA and phylo‐PCA returned 56 orthogonal vectors in the combined Carnivora + ungulates dataset. tPC1 explained 53.20% and tPC2 17.90% of the total variance (Figure 3). The first tPC described differences between orders by distinguishing between the humerus shapes of *C. antiquitatis* (extreme negative value, Perissodactyla) and *Felis margarita* (extreme positive value, Carnivora), whereas tPC2 distinguished between the humerus shapes of *Hemitragus jemlahicus* (extreme negative value, Artiodacyla), and *Ceratotherium simum* (extreme positive value, Perissodactyla). tPC1 separates species according to their degree of humerus robusticity. tPC1 values in the negative range described a thick and short humerus shape. The ventrocaudal tip of the humeral head was projected laterally while both tubercles and distal epiphyses were horizontally extended (Figure 3). In contrast, positive tPC1 values are typically associated with Carnivora with slender humerus shape and laterally compressed distal and proximal epiphyses. Deformation along tPC2 described differences between bovids and the rest of the ungulates and carnivorans. Negative tPC2 values characterize bovid‐like humerus with tubercles developed dorso‐ventrally. Positive tPC2 values described a rhinoceros‐like humerus with reduced tubercles and laterally expanded distal epiphyses (Figure 3).

A total of 44.39% of the cumulative shape variance was explained by phylo‐PC1 (36.50%) and phylo‐PC2 (7.89%). Species with humerus morphologies resembling that of the fossil bovid *Gallogoral meneghini* (Artiodactyla), with robust shape and laterally extended epiphyses, occupied phylo‐PC1 negative values, whereas species with humerus morphologies similar to *Tragelaphus spekii* (Artiodactyla), characterized by slender shape and laterally compressed epiphyses, showed positive phylo‐PC1 values (Figure 3). The second phylogenetic PC distinguished between the humerus of living *Panthera pardus* (negative values, Carnivora) with compressed epiphyses from the humerus shape of the extinct *Smilodon fatalis* (positive values, Carnivora; Figure 3) with expanded epiphyses.

Procrustes ANOVA found lnCS to be a significant predictor of humerus shape both with and without taking phylogenetic relatedness into account in the whole sample (Table 3a); however, lnCS was not significantly associated with habitat categorization (Table 3b).

**TABLE 3:** Non‐phylogenetic and phylogenetic (+phy) Procrustes ANOVA for the models with shape of living species as multivariate dependent variable and size (a) or habitat (b) as univariate independent variable.

Model	Df	SS	MS	Rsq	*F*	*Z*	*p* Value
(a)							
All							
Shape~size	1	0.296	0.296	0.248	40.156	3.849	**0.001**
Shape~size+phy	1	0.006	0.006	0.165	24.169	4.520	**0.001**
Carnivora							
Shape~size	1	0.044	0.044	0.260	13.737	3.288	**0.001**
Shape~size+phy	1	0.002	0.002	0.082	3.469	2.097	**0.017**
Ungulates							
Shape~size	1	0.215	0.215	0.315	37.319	4.013	**0.001**
Shape~size+phy	1	0.004	0.004	0.163	15.791	4.786	**0.001**
(b)							
All							
Size~habitat	2	0.399	0.199	0.025	1.179	0.519	0.310
Size~habitat+phy	2	0.023	0.012	0.034	1.606	0.836	0.201
Carnivora							
Size~habitat	2	0.135	0.068	0.025	0.369	−0.453	0.684
Size~habitat+phy	2	0.038	0.019	0.125	2.070	1.107	0.140
Ungulates							
Size~habitat	2	0.893	0.446	0.092	3.035	1.587	0.056
Size~habitat+phy	2	0.012	0.006	0.031	0.975	0.303	0.373

*Note*: *p*‐Value: probability with significance (<0.05) highlighted in bold. Size: natural log‐transformed centroid size.

Abbreviations: Df, degree of freedom; *F*, the F statistic; MS, mean squares; Rsq, coefficient of determination; SS, sums of squares; *Z*, effect‐size.

Phylogenetic signals computed for the shape variables were statistically significant except for phylo‐PCs, as expected (Table 4). Bloomberg's K was always closer to 0 than 1, except for the tPCs in the whole sample, indicating that closely related species show morphologies less similar than expected by Brownian Motion (Table 4). The categorical habitat variable has a lambda of 0.813, 0.207, and 0.933 for the whole sample, Carnivora, and ungulates, respectively.

**TABLE 4:** Phylogenetic signal (*K*) computed for size: natural log‐transformed centroid size, PCA: traditional PCA shape data; phylo‐PCA: phylogenetically corrected shape data; size‐free shape: residual shape data from the allometric model (shape~size).

	All		Carnivora		Ungulates	
	*K*	*p* Value	*K*	*p* Value	*K*	*p* Value
Size	0.359	**0.001**	0.357	**0.003**	0.458	**0.001**
tPCs	0.512	**0.001**	0.377	**0.001**	0.446	**0.001**
phylo‐PCs	0.088	1.000	0.133	0.972	0.091	1.000
Size‐free shape	0.443	**0.001**	0.368	**0.001**	0.348	**0.001**

*Note*: Significance (<0.05) highlighted in bold.

### 3.2 Habitat predictions for the whole sample

**FIGURE 4:**
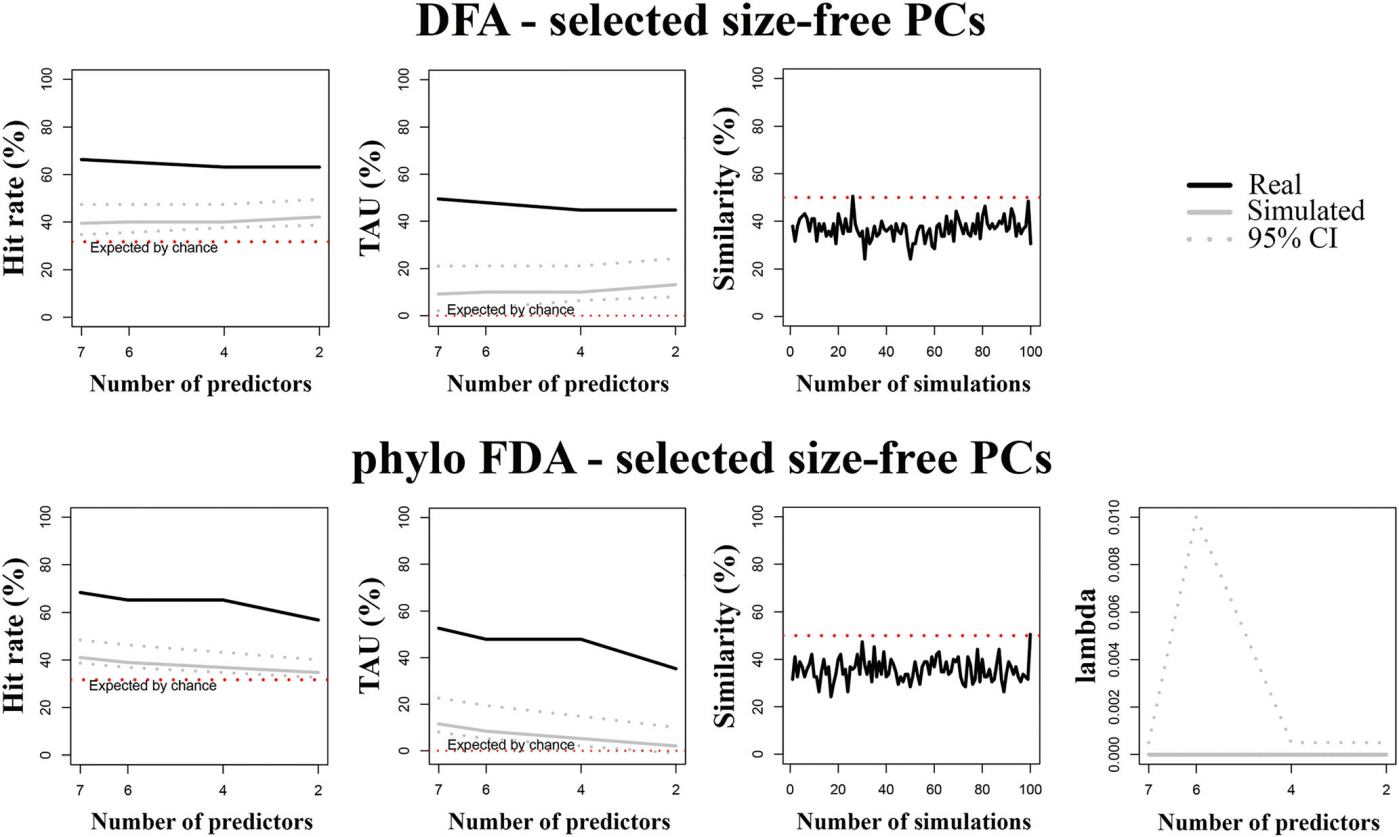
Percentage of correct classified cases (hit rate), TAU statistic and percentage of similarity (between observed and simulated habitats preferences) plotted against number of predictors for discriminant function analysis (DFA, upper) and phylogenetic Flexible Discriminant Analysis (phylo‐FDA, lower) of selected size‐free Principal Component vectors (sfPC) of the whole sample. Solid line: observed hit rate, TAU and % similarity; gray line: mean of the randomized group affiliation after 100 simulated discriminant analyses; gray dotted lines: 95th percentiles for the 100 simulated discriminant analyses with randomized group affiliation; red line: expected proportion of cases correctly classified by pure chance. In phylo‐FDA plots, lambda variation plotted against number of predictors.

Seventeen tPCs, 27 phylo‐PCs, and 20 sfPCs accounted for 95% of shape variance in the whole sample, while PGLS, which was used to identify PCs that significantly predicted habitat type, identified 6 tPCs, 6 phylo‐PCs, and 7 sfPCs. The model that performed best in the non‐phylogenetic context was the one based on selected sfPCs, which provided a hit rate of 66.32% and a TAU of 49.47% (Table 5a, Figure 4). The model also returned the highest differences from the simulated data (i.e., the highest mean differences, Table 5a).

**TABLE 5:** The highest hit rates and TAUs returned from *compare.dfa* when discriminant function analysis (a) and phylogenetic flexible discriminant analysis (b) were performed using either PCs representing 95% of total variation or selected PCs.

	No. of PCs	Hit rate	Mean diff.	*p* Value	TAU	Mean diff.	*p* Value
(a) DFA							
95% PCs							
tPCs	4	60.00	7.69	1.00	40.00	11.54	1.00
phylo‐PCs	8	43.16	−13.48	0.25	14.74	−20.22	0.25
sfPCs	4	61.05	7.78	1.00	41.58	11.67	1.00
Selected PCs							
tPCs	6	58.95	8.74	1.00	38.42	13.10	1.00
phylo‐PCs	6	52.63	−3.77	0.93	28.95	−0.55	0.93
sfPCs	**7**	**66.32**	**16.54**	**1.00**	**49.47**	**24.81**	**1.00**

	No. of PCs	Hit rate	Mean diff.	*p* Value	TAU	Mean diff.	*p* Value	Lambda
(b) Phylo‐FDA								
95% PCs								
tPCs	16	67.37	10.87	1.00	51.05	16.31	1.00	0.00
phylo‐PCs	27	62.11	−3.77	0.71	43.16	−5.66	0.71	0.10
sfPCs	20	71.58	9.57	0.96	57.37	14.35	0.96	0.00
Selected PCs								
tPCs	6	60.00	13.68	1.00	40.00	20.53	1.00	0.05
phylo‐PCs	4	48.42	2.42	0.98	22.63	3.63	0.98	0.08
sfPCs	**7**	**68.42**	**19.45**	**1.00**	**52.63**	**29.17**	**1.00**	**0.00**

*Note*: Significance of two‐tailed test for mean difference between observed and simulated data was determined when the *p*‐value exceeded 0.975. The best predictive model is in bold.

Abbreviations: DFA, discriminant function analysis; phylo‐FDA, phylogenetic flexible discriminant analysis; tPCs, traditional principal components; phylo‐PCs, phylogenetic principal components; sfPCs, size free principal components.

When *compare.dfa* was performed while accounting for the phylogenetic effect, the highest hit rate and TAU were obtained again using sfPCs accounting for 95% of the shape variance (Table 5b). However, this model was not significantly different from random simulations (*p* value = 0.96; Table 5b). Among the significant models, the model returning the highest hit rate (68.42%), TAU (52.63%), and mean differences (mean differences hit rate = 19.45, mean differences TAU = 29.17) was the one employing selected sfPCs (Table 5b, Figure 4).

**FIGURE 5:**
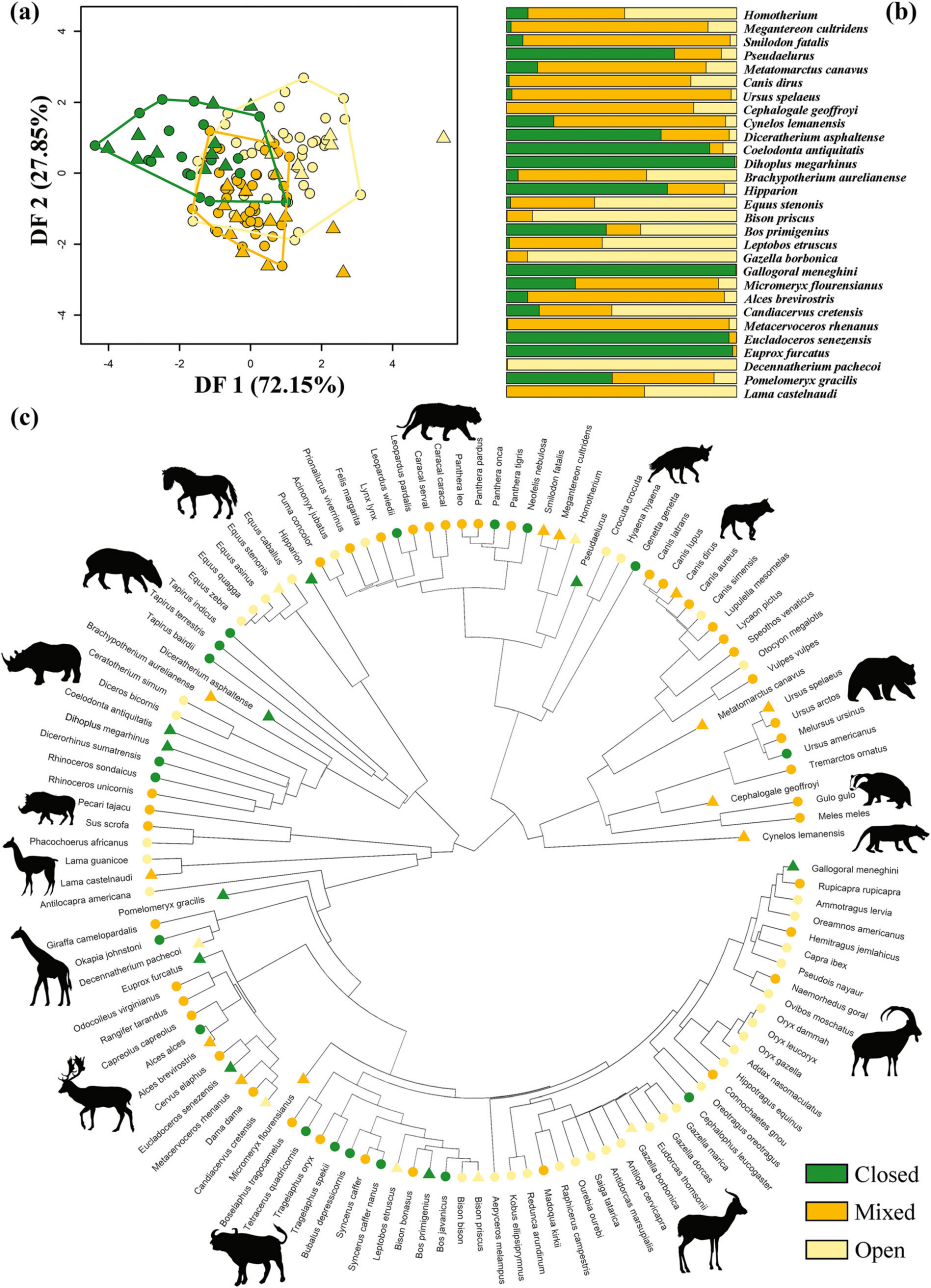
(a) Scatter plot of the first two discriminant axes (DF1 and DF2) produced by the phylo‐FDA for the entire dataset using selected size‐free PCs. (b) Bar plots of the posterior probabilities of habitat categorization for extinct species. (c) Observed and predicted habitat categories mapped within the phylogeny. Extant species are represented by circles and fossil species by triangles in (a) and (c).

The first discriminant function (72.15% of variance), returned by the best phylo‐FDA model (i.e., computed using selected sfPCs), distinguished closed‐adapted from open‐adapted species (Figure 5a). The second phylo‐DF vector (27.85% of variance) separated mixed from both closed and open habitat groups (Figure 5a). In both cases, the distinction was not clear‐cut, including a lot of overlap among groups.

The best discriminant phylogenetic model, applied to infer paleohabitat preferences in fossil species computed using selected sfPCs as predictors, found *M. cultridens*, *S. fatalis*, *M. canavus*, *C. dirus*, *U. spelaeus*, *C. geoffroyi*, and *Cynelos*. cf. *lemanensis*, among carnivorans, to prefer mixed habitats (Table 6; Figure 5b,c). *Homotherium* and *Pseudaelurus* were predicted to inhabit open and closed habitats, respectively (Table 6; Figure 5b,c). These predictions were supported by the best DFA model (i.e., using selected sfPCs; Table 6).

**TABLE 6:** Posterior probabilities of habitat categorization for fossil species based on the application of phylogenetic flexible discriminant analysis (phylo‐FDA) and discriminant function analysis (DFA) using selected size‐free PCs as predictors.

	Phylo‐FDA				DFA			
	Closed	Mixed	Open	Category	Closed	Mixed	Open	Category
*Homotherium*	9.34	41.94	48.73	**Open**	3.01	28.45	68.54	**Open**
*Megantereon cultridens*	1.95	85.55	12.49	**Mixed**	0.86	75.38	23.76	**Mixed**
*Smilodon fatalis*	7.11	90.02	2.88	**Mixed**	3.49	90.16	6.34	**Mixed**
*Pseudaelurus*	72.98	20.33	6.70	**Closed**	45.31	32.25	22.44	**Closed**
*Metatomarctus canavus*	13.50	73.18	13.32	**Mixed**	6.13	66.18	27.69	**Mixed**
*Canis dirus*	1.09	78.98	19.93	**Mixed**	0.44	64.62	34.93	**Mixed**
*Ursus spelaeus*	2.34	95.21	2.46	**Mixed**	1.15	93.50	5.35	**Mixed**
*Cephalogale geoffroyi*	0.01	81.30	18.68	**Mixed**	0.02	65.38	34.60	**Mixed**
*Cynelos* cf. *lemanensis*	20.53	74.61	4.86	**Mixed**	10.19	77.10	12.71	**Mixed**
*Diceratherium asphaltense*	67.13	29.56	3.31	Closed	38.42	48.12	13.45	Mixed
*Coelodonta antiquitatis*	88.24	5.74	6.02	**Closed**	68.50	10.00	21.50	**Closed**
*Dihoplus megarhinus*	99.16	0.80	0.04	**Closed**	97.94	1.86	0.20	**Closed**
*Brachypotherium aurelianense*	5.07	55.77	39.16	Mixed	1.83	40.27	57.90	Open
*Hipparion*	69.84	24.75	5.40	**Closed**	46.07	36.80	17.13	**Closed**
*Equus stenonis*	1.78	36.46	61.76	**Open**	0.52	22.09	77.38	**Open**
*Bison priscus*	0.27	10.94	88.79	**Open**	0.07	5.74	94.19	**Open**
*Bos primigenius*	43.34	14.95	41.71	Closed	16.52	12.53	70.95	Open
*Leptobos etruscus*	1.24	40.34	58.42	**Open**	0.37	24.85	74.78	**Open**
*Gazella borbonica*	0.31	8.75	90.94	**Open**	0.08	4.74	95.18	**Open**
*Gallogoral meneghini*	99.65	0.34	0.00	**Closed**	99.12	0.85	0.03	**Closed**
*Micromeryx flourensianus*	29.92	62.14	7.94	**Mixed**	14.56	66.08	19.35	**Mixed**
*Alces brevirostris*	9.21	85.39	5.40	**Mixed**	4.42	84.01	11.58	**Mixed**
*Candiacervus cretensis*	14.21	31.52	54.27	**Open**	4.45	21.05	74.50	**Open**
*Metacervocerus rhenanus*	0.51	96.16	3.33	**Mixed**	0.25	92.63	7.12	**Mixed**
*Eucladoceros senezensis*	96.63	3.22	0.16	**Closed**	92.39	6.88	0.73	**Closed**
*Euprox furcatus*	98.20	1.76	0.04	**Closed**	94.77	4.94	0.29	**Closed**
*Decennatherium pachecoi*	0.00	0.44	99.56	**Open**	0.00	0.33	99.67	**Open**
*Pomelomeryx gracilis*	45.92	44.19	9.89	Closed	21.76	52.10	26.14	Mixed
*Lama castelnaudi*	0.07	59.87	40.06	Mixed	0.03	41.37	58.60	Open

*Note*: In bold, match between phylo‐FDA and DFA inferences.

phylo‐FDA predicted *D. asphaltense*, *C. antiquitatis*, *D. megarhinus*, and *Hipparion* to likely live in closed habitats (Table 6; Figure 5b,c). Among the other Perissodactyla, *E. stenonis* was predicted to prefer open habitats while mixed vegetation was predicted for *B. aurelianense* (Table 6; Figure 5b,c). These predictions were not entirely consistent with the best DFA model results, which classified *D. asphaltense* as mixed and *B. aurelianense* as open‐adapted (Table 6).

Among the Artiodactyla, phylo‐FDA predicted open habitats for *L. etruscus*, *B. priscus*, *G. borbonica*, *C. cretensis*, and *D. pachecoi* (Table 6 and Figure 5b,c) and closed habitats for *B. primigenius*, *G. meneghini*, *E. senezensis*, *P. gracilis*, and *E. furcatus*. While *M. flourensianus*, *A. brevirostris*, *M. rhenanus*, and *L. castelnaudi* were classified as mixed species (Table 6 and Figure 5b,c). The best DFA model results largely supported these predictions except for *B. primigenius*, *L. castelnaudi* (both predicted as open), and *P. gracilis* (considered mixed by nonphylogenetic DFA; Table 6).

### 3.3 Habitat predictions for the Carnivora

**FIGURE 6:**
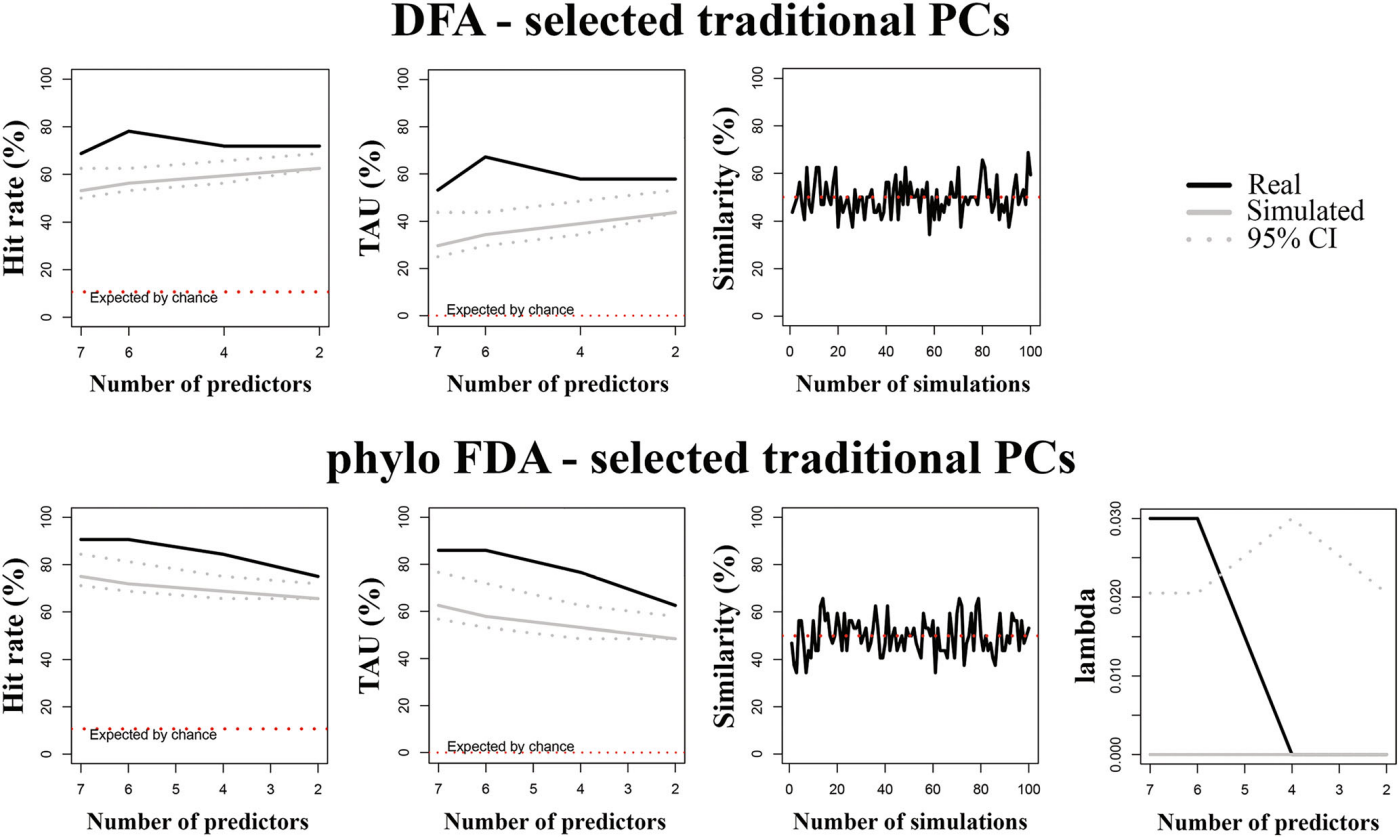
Percentage of correct classified cases (hit rate), TAU statistic and percentage of similarity (between observed and simulated habitats preferences) plotted against number of predictors for discriminant function analysis (DFA, upper) and phylogenetic Flexible Discriminant Analysis (phylo‐FDA, lower) of selected traditional Principal Component vectors (tPCs) of the Carnivora sample. Solid line: observed hit rate, TAU and % similarity; gray line: mean of the randomized group affiliation after 100 simulated discriminant analyses; gray dotted lines: 95th percentiles for the 100 simulated discriminant analyses with randomized group affiliation; red line: expected proportion of cases correctly classified by pure chance. In phylo‐FDA plots, lambda variation plotted against number of predictors.

In the Carnivora, 13 tPCs, 15 phylo‐PCs, and 15 sfPCA explained 95% of the shape variance, while 7, 5, and 6 PCs from tPCs, phylo‐PCs, and sfPCs subsets, respectively, were selected by PGLS. *compare.dfa* function showed that non‐phylogenetic models based on selected PCs performed better than models using 95% PCs (Table 7a). Specifically, the best DFA model was the one using 6 selected tPCs (out of 7 selected PCs; Table 7a; Figure 6) which returned the highest metric (hit rate = 78.13%, TAU = 67.19%) and the highest mean differences (mean differences hit rate = 7.81, mean differences TAU = 11.72).

**TABLE 7:** The highest hit rates and TAUs returned from *compare.dfa* when discriminant function analysis (a) and phylogenetic flexible discriminant analysis (b) were performed using either PCs representing 95% of total variation or selected PCs.

	No. of PCs	Hit rate	Mean diff.	*p* Value	TAU	Mean diff.	*p* Value
(a) DFA							
95% PCs							
tPCs	2	65.63	−4.51	0.78	48.44	−6.76	0.78
phylo‐PCs	2	62.50	−10.98	0.50	43.75	−16.46	0.50
sfPCs	15	68.75	−3.16	0.71	53.13	−4.75	0.71
Selected PCs							
tPCs	**6**	**78.13**	**7.81**	**1.00**	**67.19**	**11.72**	**1.00**
phylo‐PCs	2	71.88	0.00	0.97	57.81	0.00	0.97
sfPCs	4	75.00	7.29	1.00	62.50	10.94	1.00

	No. of PCs	Hit rate	Mean diff.	*p* Value	TAU	Mean diff.	*p* Value	Lambda
(b) phylo‐FDA								
95% PCs								
tPCs	13	93.75	−4.46	0.68	90.63	−6.70	0.68	0.00
phylo‐PCs	14	93.75	−4.75	0.66	90.63	−7.12	0.66	0.00
sfPCs	14	96.88	−3.55	0.74	95.31	−5.33	0.74	0.00
Selected PCs								
tPCs	**7**	**90.63**	**6.25**	**0.99**	**85.94**	**9.38**	**0.99**	**0.03**
phylo‐PCs	5	84.38	3.07	0.96	76.56	4.61	0.96	0.00
sfPCs	6	87.50	4.17	0.95	81.25	6.25	0.95	0.00

*Note*: Significance of two‐tailed test for mean difference between observed and simulated data was determined when the *p*‐value exceeded 0.975. The best predictive model is in bold.

Abbreviations: DFA, discriminant function analysis; phylo‐FDA, phylogenetic flexible discriminant analysis; phylo‐PCs, phylogenetic principal components; sfPCs, size free principal components; tPCs, traditional principal components.

Although the phylo‐FDA models with the highest hit rate and TAU were the ones based on PCs expressing 95% of the variance, their expectations were not significantly different from random simulated data (Table 7b). The model with selected tPCs appears to have the highest hit rate and TAU outperforming simulated data (Table 7b; Figure 6).

**FIGURE 7:**
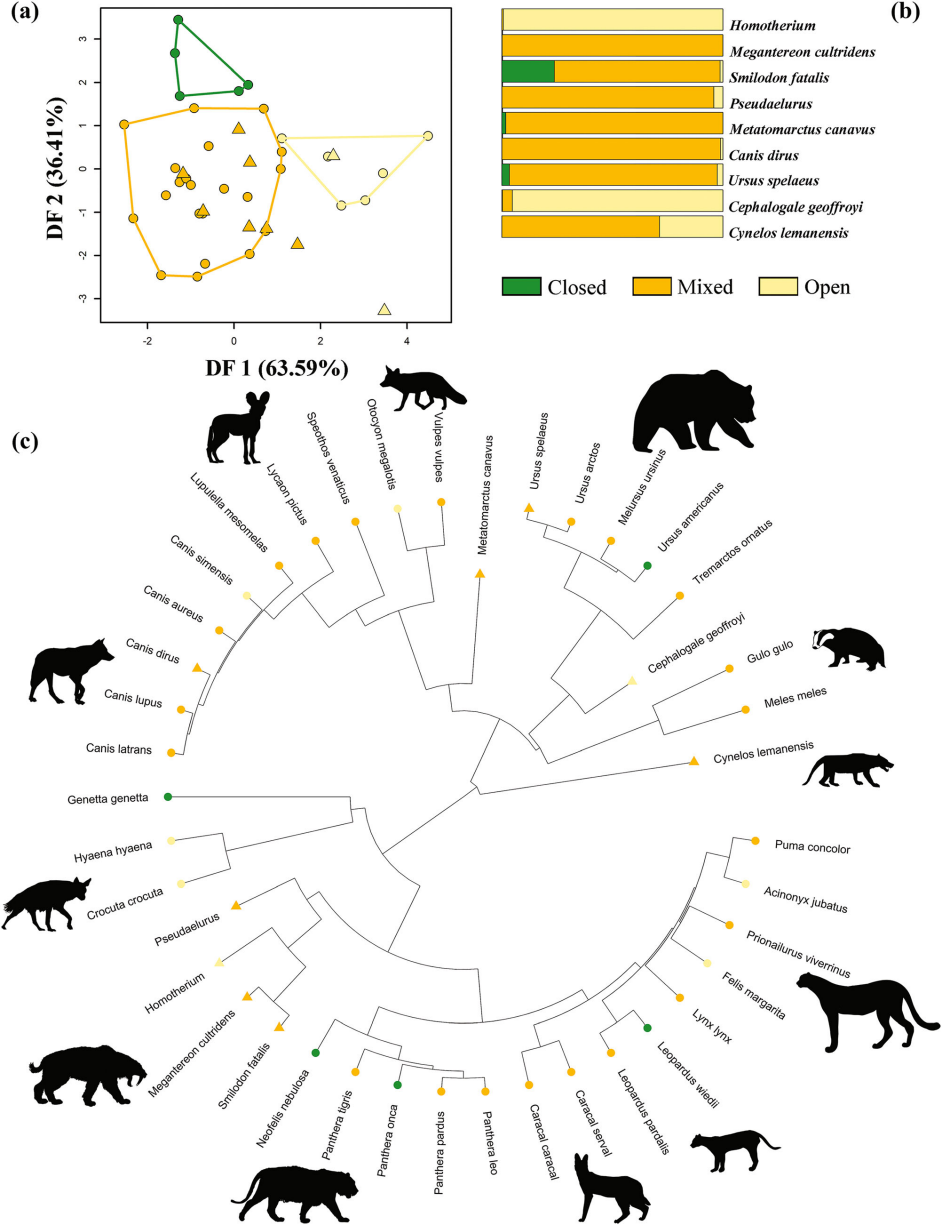
(a) Scatter plot of the first two discriminant axes (DF1 and DF2) produced by the phylo‐FDA for the Carnivora dataset using selected traditional PCs. (b) Bar plots showing the posterior probabilities of habitat categorization obtained for extinct species. (c) The observed and predicted habitat categories are mapped within Carnivora phylogeny. Extant species are represented by circles and fossil species by triangles in (a) and (c).

DF1 (63.59% of variance) of the best phylogenetic model, based on selected tPCs as predictors, discriminated species preferring both closed and mixed vegetation types from species preferring open habitats. DF2 (36.41% of variance) distinguished between both mixed and open‐adapted species from the closed ones (Figure 7a). In this case, the estimated lambda was 0.03.

phylo‐FDA predicted *Homotherium* and *C. geoffroyi* in the open category, while *M. cultridens*, *S. fatalis*, *Pseudaelurus*, *M. canavus*, *C. dirus*, *U. spelaeus*, and *Cynelos*. cf. *lemanensis* in mixed habitats (Table 8 and Figure 7b,c). Predictions made using the best phylo‐FDA model matched with the predictions made using the same model without accounting for the phylogenetic correction. There was only one exception regarding *S. fatalis* which in non‐phylogenetic analysis was projected in the closed habitat (Table 8).

**TABLE 8:** Posterior probabilities of habitat categorization for fossil species based on the application of phylogenetic flexible discriminant analysis (phylo‐FDA) and discriminant function analysis (DFA) using selected traditional PCs as predictors.

	Phylo‐FDA				DFA			
	Closed	Mixed	Open	Category	Closed	Mixed	Open	Category
*Homotherium*	0.00	0.63	99.37	**Open**	0.00	1.95	98.05	**Open**
*Megantereon cultridens*	0.15	99.81	0.04	**Mixed**	0.65	99.28	0.07	**Mixed**
*Smilodon fatalis*	23.70	74.97	1.33	Mixed	65.10	34.11	0.79	Closed
*Pseudaelurus*	0.05	95.81	4.15	**Mixed**	0.49	97.76	1.75	**Mixed**
*Metatomarctus canavus*	1.77	98.22	0.01	**Mixed**	0.45	99.39	0.16	**Mixed**
*Canis dirus*	0.05	98.83	1.12	**Mixed**	0.02	95.56	4.42	**Mixed**
*Ursus spelaeus*	3.40	93.98	2.61	**Mixed**	3.44	90.83	5.74	**Mixed**
*Cephalogale geoffroyi*	0.24	4.47	95.30	**Open**	0.17	9.22	90.61	**Open**
*Cynelos* cf*. lemanensis*	0.01	71.29	28.69	**Mixed**	0.09	82.79	17.12	**Mixed**

*Note*: In bold, match between phylo‐FDA and DFA inferences.

### 3.4 Habitat predictions for ungulates

**FIGURE 8:**
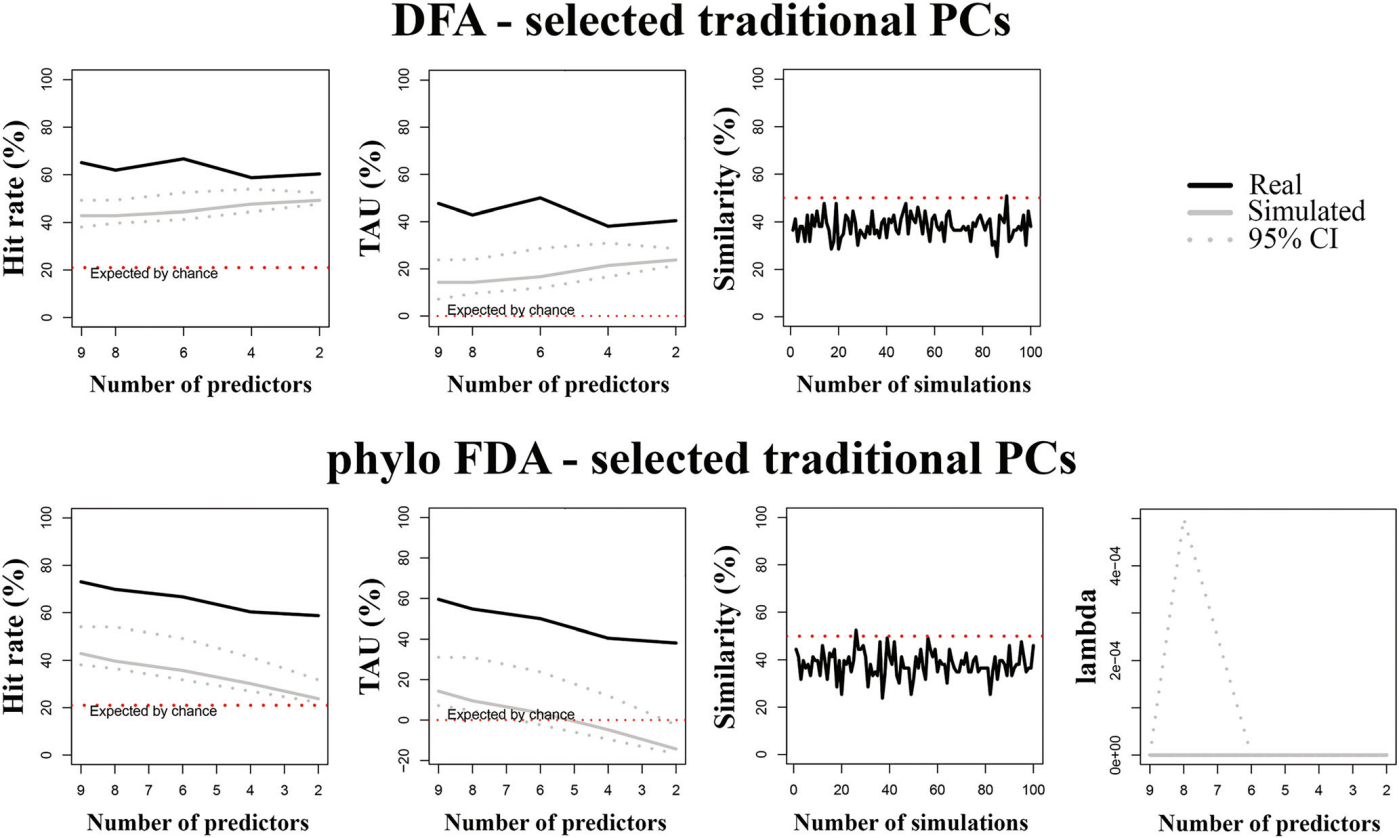
Percentage of correct classified cases (hit rate), TAU statistic and percentage of similarity (between observed and simulated habitats preferences) plotted against number of predictors for discriminant function analysis (DFA, upper) and phylogenetic Flexible Discriminant Analysis (phylo‐FDA, lower) of selected traditional Principal Component vectors (tPCs) of the ungulates sample. Solid line: observed hit rate, TAU and % similarity; gray line: mean of the randomized group affiliation after 100 simulated discriminant analyses; gray dotted lines: 95th percentiles for the 100 simulated discriminant analyses with randomized group affiliation; red line: expected proportion of cases correctly classified by pure chance. In phylo‐FDA plots, lambda variation plotted against number of predictors.

In the ungulates sample, 18 tPCs, 25 phylo‐PCs, and 22 sfPCs described 95% of the shape variance, while 9 tPCs, 7 phylo‐PCs, and 7 sfPCs were selected by the PGLS method and they significantly predicted habitat categories. The model returning the highest performance metrics was the one employing 6 tPCs (out of 9 selected PCs; Table 9a and Figure 8).

**TABLE 9:** The highest hit rates and TAUs returned from *compare.dfa* when discriminant function analysis (a) and phylogenetic flexible discriminant analysis (b) were performed using either PCs representing 95% of total variation or selected PCs.

	No. of PCs	Hit rate	Mean diff.	*p* Value	TAU	Mean diff.	*p* Value
(a) DFA							
95% PCs							
tPCs	4	58.73	−0.37	0.88	38.10	−0.56	0.88
phylo‐PCs	2	49.21	−11.76	0.37	23.81	−17.65	0.37
sfPCs	6	57.14	1.98	0.95	35.71	2.97	0.95
Selected PCs							
tPCs	**6**	**66.67**	**11.08**	**1.00**	**50.00**	**16.62**	**1.00**
phylo‐PCs	7	57.14	1.19	0.94	35.71	1.79	1.94
sfPCs	2	61.90	7.50	1.00	42.86	11.25	1.00

	No. of PCs	Hit rate	Mean diff.	*p* Value	TAU	Mean diff.	*p* Value	Lambda
(b) phylo‐FDA								
95% PCs								
tPCs	18	77.78	6.14	0.96	66.67	9.21	0.96	0.00
phylo‐PCs	25	73.02	−8.10	0.62	59.52	−12.14	0.62	0.02
sfPCs	20	73.02	2.29	0.90	59.52	3.43	0.90	0.00
Selected PCs								
tPCs	**9**	**73.02**	**21.52**	**1.00**	**59.52**	**32.29**	**1.00**	**0.00**
phylo‐PCs	7	58.73	3.55	0.93	38.10	5.33	0.93	0.03
sfPCs	7	60.32	11.07	1.00	40.48	16.61	1.00	0.00

*Note*: Significance of two‐tailed test for mean difference between observed and simulated data was determined when the *p*‐value exceeded 0.975. The best predictive model is in bold.

Abbreviations: DFA, discriminant function analysis; phylo‐FDA, phylogenetic flexible discriminant analysis; phylo‐PCs, phylogenetic principal components; sfPCs, size free principal components; tPCs, traditional principal components.

*compare.dfa* computed in a phylogenetic context showed that the observed data significantly differed from the simulated data only when selected PCs were employed (Table 9b). When using selected tPCs, classification rates exceeded 70% and TAU was close to 60% while returning the highest mean differences (Table 9b, Figure 8).

**FIGURE 9:**
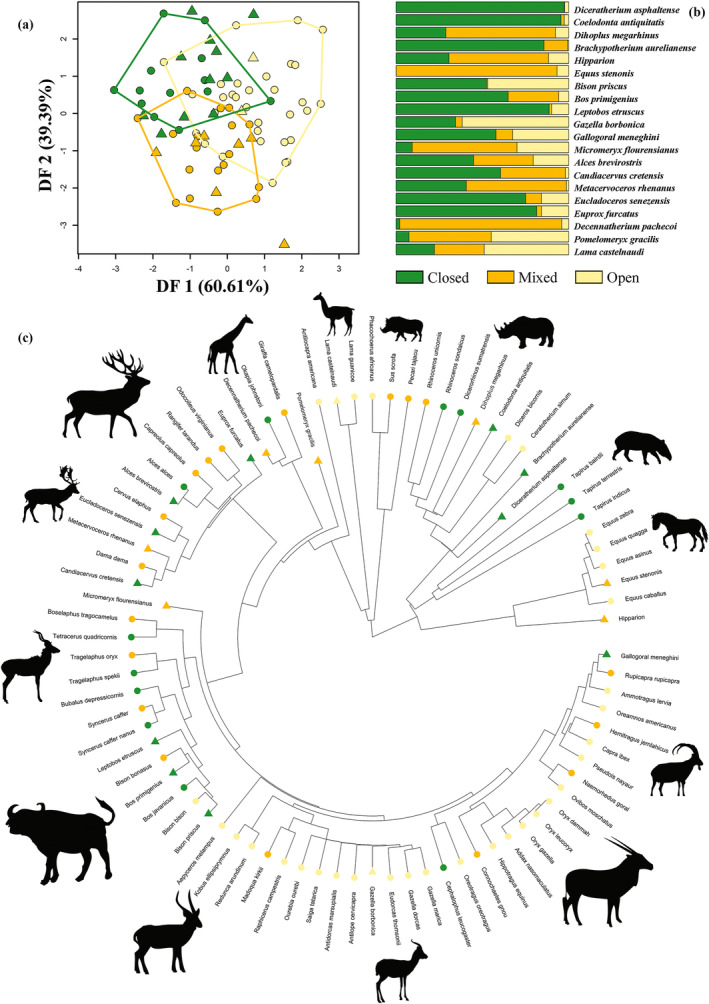
(a) Scatter plot of the first two discriminant axes (DF1 and DF2) returned from the phylo‐FDA computed on ungulates using selected traditional PCs. (b) Bar plots of the posterior probabilities returned for fossil species. (c) The observed and predicted habitat categories mapped within ungulates phylogeny. Extant species are represented by circles and fossil species by triangles in (a) and (c).

The resulting DF axes of the phylo‐FDA analysis computed using selected tPCs showed some degree of overlap between habitat categories (Figure 9a). DF1 (60.61% of variance) distinguished between species preferring both mixed and closed vegetation from species preferring open vegetation, while DF2 (39.39% of variance) distinguished both closed and open groups from the mixed one (Figure 9a). Pagel's lambda was equal to zero.

The best selected model was phylo‐FDA using 9 selected tPCs (Table 9b). This model projected *D. asphaltense*, *C. antiquitatis*, and *B. aurelianense* in the closed category, while *D. megarhinus*, *Hipparion*, and *E. stenonis* were predicted in the mixed category (Table 10; Figure 9b,c). The non‐phylogenetic DFA analyses supported these predictions (Table 10).

**TABLE 10:** Posterior probabilities of habitat categorization for fossil species based on the application of phylogenetic flexible discriminant analysis (phylo‐FDA) and discriminant function analysis (DFA) using selected traditional PCs as predictors.

	Phylo‐FDA				DFA			
	Closed	Mixed	Open	Category	Closed	Mixed	Open	Category
*Diceratherium asphaltense*	97.39	0.24	2.38	**Closed**	77.47	1.30	21.23	**Closed**
*Coelodonta antiquitatis*	95.50	1.85	2.65	**Closed**	80.78	4.34	14.88	**Closed**
*Dihoplus megarhinus*	28.78	63.52	7.70	**Mixed**	12.75	66.12	21.13	**Mixed**
*Brachypotherium aurelianense*	85.64	13.65	0.71	**Closed**	66.41	29.10	4.48	**Closed**
*Hipparion*	30.59	57.72	11.69	**Mixed**	12.82	56.82	30.36	**Mixed**
*Equus stenonis*	0.03	93.22	6.75	**Mixed**	0.01	83.68	16.31	**Mixed**
*Bison priscus*	52.37	0.65	46.98	Closed	15.63	0.55	83.83	Open
*Bos primigenius*	64.90	29.28	5.82	Closed	37.11	41.66	21.23	Mixed
*Leptobos etruscus*	88.64	1.45	9.91	**Closed**	56.90	2.60	40.50	**Closed**
*Gazella borbonica*	34.42	3.84	61.74	**Open**	8.55	2.58	88.87	**Open**
*Gallogoral meneghini*	57.79	9.71	32.50	Closed	20.54	9.13	70.33	Open
*Micromeryx flourensianus*	9.32	60.73	29.95	Mixed	3.22	42.23	54.55	Open
*Alces brevirostris*	44.87	34.53	20.61	Closed	17.62	33.23	49.14	Open
*Candiacervus cretensis*	60.47	37.61	1.92	Closed	36.54	55.81	7.65	Mixed
*Metacervocerus rhenanus*	40.67	58.03	1.31	**Mixed**	21.67	73.76	4.57	**Mixed**
*Eucladoceros senezensis*	75.02	9.14	15.84	Closed	37.96	12.08	49.96	Open
*Euprox furcatus*	81.46	2.74	15.80	Closed	41.15	4.50	54.35	Open
*Decennatherium pachecoi*	2.09	93.83	4.08	**Mixed**	1.13	86.58	12.28	**Mixed**
*Pomelomeryx gracilis*	7.44	47.68	44.89	Mixed	2.51	29.40	68.09	Open
*Lama castelnaudi*	22.17	28.81	49.02	**Open**	5.93	18.56	75.51	**Open**

*Note*: In bold, match between phylo‐FDA and DFA inferences.

All fossil bovids were predicted to live in closed vegetation (Table 10; Figure 9b,c). These results were not supported by using the best non‐phylogenetic DFA model which predicted *B. priscus* and *L. etruscus* to prefer open and mixed habitats, respectively (Table 10).

With respect to the remaining Artiodactyla species, *G. borbonica* and *L. castelanaudi* were placed in open category while *G. meneghini*, *A. brevirostris*, *C. cretensis*, *E. senezensis*, and *E. furcatus* were in closed (Table 10; Figure 9b,c). *M. flourensianus*, *M. rhenanus*, *D. pachecoi*, and *P. gracilis* were in the morphospace region of the mixed‐adapted species (Table 10; Figure 9b,c). When using the best DFA model based on nine tPCs only the predictions made for *G. borbonica*, *M. rhenanus*, *D. pachecoi*, and *L. castelanaudi* matched with the phylo‐FDA predictions (Table 10).

## 4 DISCUSSION

This study shows that humerus morphology of Carnivora and ungulates can be used to distinguish species according to their preferred environmental adaptations thus allowing the prediction of paleohabitat for fossil species. The statistical model based on the whole species sample provided an accuracy of over 65% with size free shape data being selected as the best predictors accounting for phylogenetic relatedness. This percentage was estimated comparing our predictions with other palaeoecological reconstruction found in literature (see Section 1.1 for detail). Considering the strong biomechanical constrain imposed by body mass on humerus morphology of large mammals this result supports previous observations on bone shape changes which occur at large body sizes generally attained by ungulates when analyzed in conjunction with carnivorans (Bertram & Biewener, 1990).

Extraction of shape data using traditional PCA was effective for both Carnivora and ungulates when clades were analyzed individually. As previously suggested by Meloro et al. (2013) and DeGusta and Vrba (2003), less transformed data (i.e., traditional shape data) provided more accurate paleoenvironmental reconstructions than more transformed one (i.e., both size and phylogenetic free shape) especially when focusing on a range of taxa with similar morphological bauplan.

Use of subsets of selected PCs additionally produced better predictive models even if they did not return the highest hit rates and TAUs. This finding may be explained by the plasticity of the humerus shape whose anatomical features are driven by different selective pressures broadly captured by each specific PC axis. This has also already been noted for bovids (Barr & Scott, 2014) and we recommend the selection of shape components which are significantly correlated with the ecological variable under investigation, to improve the power of predictive models.

**FIGURE 10:**
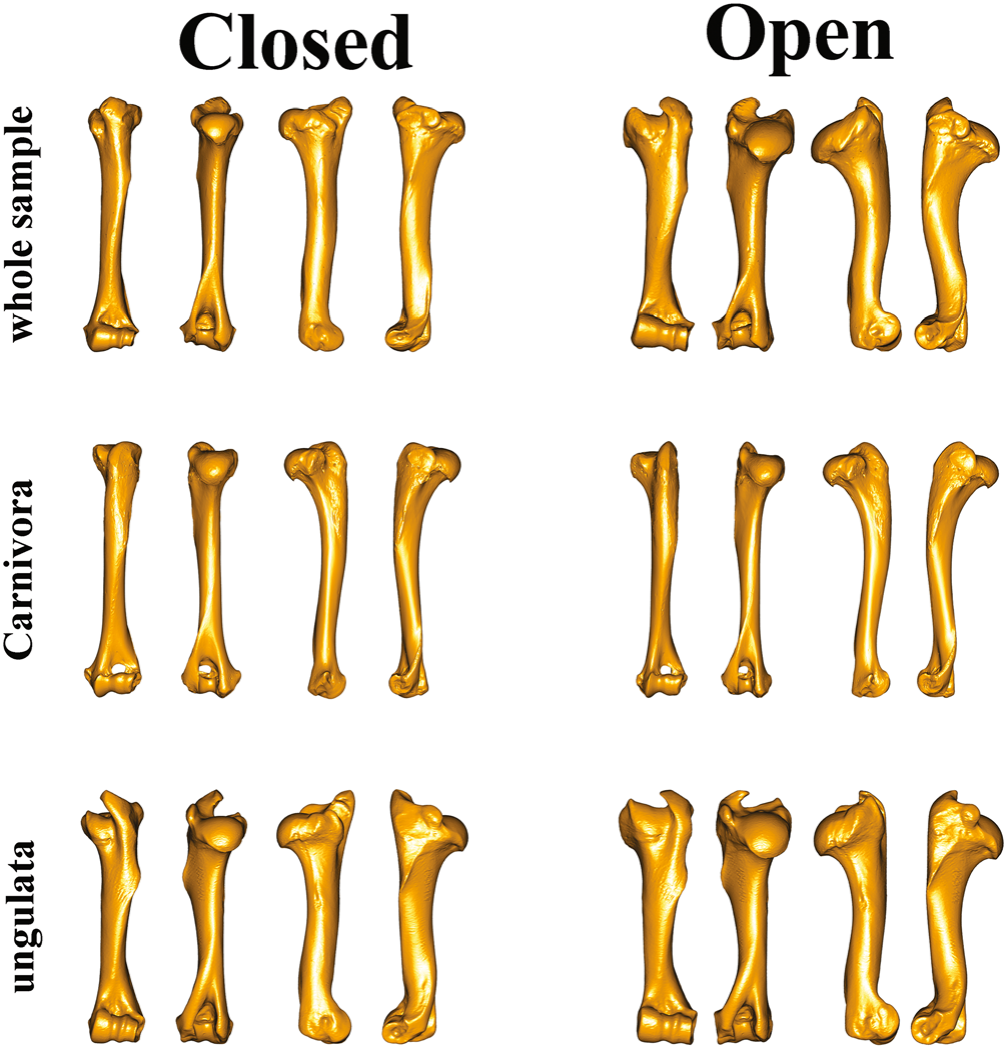
Humerus shape deformation restored from discriminant functions separating Closed from Open adapted species for the whole sample (*n* = 94), Carnivora (*n* = 32) and ungulates (*n* = 63). Reference specimen for the whole sample was *Cervus elaphus NMB‐Pf.118*, for Carnivora *Canis lupus* HNHM.V.58.1735, for ungulates *Hippotragus equinus* MNHN‐ZM‐AC‐1969‐167.

All three analyzed datasets (i.e., the whole sample, Carnivora, and ungulates) shared the same morphological predictors gradually changing from closed to open vegetation cover (Figure 10). Species living in closed vegetation have a humerus with compressed epiphyses and a triangular humeral head. The subscapularis muscle, which stabilizes the shoulder articulation and contribute to the flexion and extension of the shoulder joint, is attached to the lesser tubercle (Barone, 1999; Janis & Figueirido, 2014). These characteristics imply that species living in closed habitats have less constrained articulations and can change direction when moving with greater ease. By contrast, the humerus in species associated with open vegetation types generally showed laterally expanded epiphyses. The greater tubercle is more expanded dorsally while the humeral head is wider caudally. The greater tuberosity is the insertion point of the supraspinatus and infraspinatus muscles. These muscles belong to the rotator cuff complex and take part in the stabilization of the shoulder (Barone, 1999; Janis & Figueirido, 2014). The general pattern we observed suggests that the morphologies of species living in open vegetation type evolved to stabilize the articulations, increase propulsion during fast running or long walking, and constrain forearm movement to the parasagittal plane. These cross‐taxa findings generalize on previous patterns already observed within single orders (Etienne et al., 2021; Janis & Figueirido, 2014; Kappelman et al., 1997; Martín‐Serra et al., 2016) suggesting that the evolution of convergent morphological traits in humerus morphology is derived from similar selective pressures.

The results of this work support the idea that the humerus is a complex anatomical structure whose shape interspecific variation is constrained between phylogeny, ecology, and body size. The fact that analyses using phylogenetic shape variables (i.e., variables from which we removed the phylogenetic component affecting interspecific shape variation) produced worse results in almost all cases and that phylogenetic‐informed FDA consistently outperformed DFA could indicate that ecological adaptations are phylogenetically nested. This is supported by our lambda statistics which demonstrated that closely related species share similar habitat preferences, a pattern particularly relevant for both the whole sample and the ungulates.

Analyses based on size free shape data always performed better than those based on the phylogenetically corrected ones. However, humerus size is not associated with habitat adaptations. Within Artiodactyla, for example, species with different body sizes were found to be distributed homogeneously among different habitats (Klein et al., 2010). Still allometric variation might obscure subtle adaptations in humerus shape data. Larger species shared enlarged epiphyses and more robust humeri relative to the slender humeri and reduced epiphyses found in small species (Etienne et al., 2021; Mallet et al., 2019; Martín‐Serra et al., 2014).

The phylogenetic signal test returned significant results indicating that closely related species exhibit similar morphologies. In all cases, we found closely related species to be less similar than expected under the Brownian Motion model of evolution. This result is consistent with other geometric morphometric studies on mammalian postcranial bones (Etienne et al., 2020, 2021; Fabre et al., 2015; Lewton et al., 2020; Püschel & Sellers, 2016; San Millán et al., 2015), hence this might be considered as a generalized pattern at least among mammals.

Orders are well separated in the traditional morphospace, supporting the strong phylogenetic signal in the humerus shape data. The axis of maximum variation separated the robust woolly rhinoceros morphology from the slender humerus of the smaller sand cat by virtue of differences in body size (Martín‐Serra et al., 2014). Other studies have highlighted slenderness as an adaptation to cursoriality (Janis & Wilhelm, 1993; Samuels et al., 2013; Stein & Casinos, 1997; Taylor, 1989; Van Valkenburgh, 1987). In the rhinoceros, the relatively robust humerus shows an increase of the articulation surface to dissipate forces (Jenkins, 1973; Mallet et al., 2019). Artiodactyla, which in the morphospace are positioned between Carnivora and Perissodactyla, showed intermediate morphology: slender humerus with enlarged epiphyses. This pattern was already observed in bovids by Etienne et al. (2021).

Phylogenetic FDA prediction, made using the whole sample, classified most of the extinct Carnivora as mixed habitat‐adapted species. This corroborates previous observations that supported a generalized forelimb morphology for predators adapted to different locomotory strategies and foraging techniques (Samuels et al., 2013; Van Valkenburgh, 1987), while the broader diversity of habitat predictions in ungulates is possibly the result of stronger selective adaptations to optimize locomotion in terrestrial environments only.

Focusing on the fossil carnivoran species, the habitat of *M. cultridens* has been subject to diverse opinions. According to Christiansen and Adolfssen (2007), *M. cultridens* was an open habitat‐adapted species. In contrast, Meloro (2011) inferred adaptations to a tropical biome based on the brachial index. These results may indicate that *Megantereon* was a species with a flexible ecological adaptation that might have favored a variety of habitats to optimize its ambushing technique (Lewis & Werdelin, 2010; Li & Sun, 2022). Similar discussions have prevailed for other species such as the cave bear (*U. spelaeus*). Open environments may have been the most likely habitat for *U. spelaeus* (Meloro & de Oliveira, 2019). However, Bocherens et al. (1994) inferred from the tooth collagen 13C levels that this extinct bear lived in forested environments. Our prediction as “mixed” species is coherently identified also by the analysis of the Carnivora subsample and implies a broad degree of adaptability for this generalist taxon.

For the commonest predators of the California site of Rancho La Brea (*C. dirus* and *S. fatalis*) a mixed environment was generally predicted by all the DFA models except in one case with *Smilodon* being classified as closed. This result is coherent with Meloro et al. (2013) findings on the larger *S. populator* and is potentially the result of the strong selective pressure that hunting mode exerted on this sabertooth cat. The humerus of *Smilodon* species was much more robust than the one of living panthers of comparable size (Meachen‐Samuels & Van Valkenburgh, 2010).

The Miocene Borophaginae dog *M. canavus* was also found in present‐day California and it had a broad distribution (Wang & Tedford, 2008). Because of its widespread distribution, this species was likely to inhabit a variety of habitats, supporting our prediction of a mixed‐habitat taxon that equally aligns with its relatively small size compared to that of more derived Borophaginae.

The whole sample prediction assigned *Pseudaelurus* to closed habitat as already suggested by Domingo et al. (2017) although the subsample DFA result categorized this taxon as mixed. *Pseudaelurus* coexisted with larger predators (Domingo et al., 2017) and due to resource partitioning and prey size selection, it may have sought refuge in wooded areas, avoiding direct encounters with larger predators (Durant, 1998). Rothwell (2001) described *Pseudaeulurus* postcranial proportion as quite unique among felids, hence palaeoecological reconstructions based on living comparative samples might be more difficult to validate.

By contrast, *Homotherium* was clearly found to prefer open habitats in all the analyses, supporting results of Meloro (2011). *Homotherium* shows a combination of dental characters that appear adapted to preying on open‐habitat species while hind limb morphology revealed cursorial adaptation typical of species living in grassland (Antón et al., 2005; DeSantis et al., 2021; Meloro, 2011).

Among Perissodactyla, *C. antiquitatis* and *D. asphaltense* were predicted to inhabit closed habitats. *C. antiquitatis* was also known to be a grazer inhabiting open‐steppe environments (Stefaniak et al., 2021), however, its diet likely included woody materials suggesting that this grassland species potentially inhabited forests, too (Tiunov & Kirillova, 2010). According to a previous study, the extinct *D. asphaltense* inhabited swampy areas and wetlands near riverine grasslands (Antoine & Becker, 2013). The conspecific *B. aurelianense* was classified to live in mixed environments, a very likely prediction also supported by its tooth morphology typical of a mixed feeder living areas with medium/high tree cover (Rafeh et al., 2020). *E. stenonis* was predicted to prefer an open habitat and the genus *Hipparion* a closed habitat. While *Equus* is classically interpreted open grassland species, the study of the hypsodonty index showed their ancestors to be adapted to a broader range of habitats from closed forests to open grasslands (Stefaniak et al., 2021).

Among large bovids, the steppe bison (*B. priscus*) and *L. etruscus* were classified as open habitat‐adapted species supporting previous palaeoecological studies (Bocherens et al., 2015; Strani et al., 2018). Analyses on the aurochs (*B. primigenius*) tooth microwear showed this bovid was capable of feeding on leaves and trees suggesting it might have inhabited marginal habitats of forested areas (Mead et al., 2014; Schulz & KaiSer, 2007) supporting our inferences.

*G. borbonica* and *G. meneghini*, which were found in the same deposits in Central Italy, were assigned to open and closed habitats, respectively. Palaeoecological reconstructions have revealed both species to adopt different feeding strategies for avoiding competition, which might equally support their habitat partitioning (Bellucci & Sardella, 2015; Eastham et al., 2016; Strani et al., 2015). *M. flourensianus* and *E. furcatus* from the site of Steinhem (Germany) were assigned by our analyses into categories which conflict with previous reconstructions: *M. flourensianus* as mixed [*contra* closed predicted by Aiglstorfer et al. (2014) and Eastham et al. (2016)] and *E. furcatus* as closed (Aiglstorfer et al., 2014). The paleoenvironment of Steinhem was described by Tütken et al. (2006) as warm‐temperate with high‐humidity which supports the idea of abundant forest canopy. It might be likely that both species shifted their preferred habitat adaptation to avoid competition over browsing. Paleoenvironmental reconstruction of Ceyssaguet, where the fossil remains of the cervid *M. rhenanus* were found, showed that the site was open grassland with wooded habitats near the lake, under cold climate (Kaiser & Croitor, 2004). This finding equally contrasts with our results which predicted *Metacervocerus* to prefer mixed habitats. However for *E. senezensis* we identified closed habitats adaptations in line with paleoenvironmental reconstruction of its fossiliferous sites (Berlioz et al., 2018; Kaiser & Croitor, 2004). The closed habitat preferences inferred for *C. cretensis* disagree with the previous inferences based on its fossil morphology (Caloi & Palombo, 1996; de Vos, 2000) while Agustí and Antón (2002) supported our prediction for the giraffid *D. pachecoi* that preferred open environments. Our analyses also classified *C. geoffroyi* and *Cynelos* cf. *lemanensis*, which were found in the same site of Allier in France, to prefer mixed habitats; *D. megarhinus* was estimated to prefer closed habitats as *P. gracilis*, while *A. brevirostris* and *L. castelnaudi* were projected in mixed habitats.

There is no doubt that palaeoecological reconstructions require a multidisciplinary approach and our DFA analyses were limited by several factors which include: (1) the environmental plasticity of large mammals whose habitat selection might be affected by multiple behavioral and environmental factors (Morris, 2003); (2) limitation in the taxonomic coverage of the groups investigated that might not include the whole ecological diversity exhibited by large mammalian predators and prey; (3) the many‐to‐one mapping of form to function that generally prevents ecological convergence to be identified [i.e., species evolve multiple morphological optima for the same ecological adaptation, Tamagnini et al., 2021].

As such our predictions should be considered with caution and validated with other proxies that can be eventually applied within more specific stratigraphical and geographical contexts (e.g., see White et al., 2009).

In this study, we also assessed multiple predictive techniques including the linear discriminant analysis (Kappelman, 1988, 1991; Kovarovic & Andrews, 2007; Scott et al., 1999) and its phylogenetically informed counterpart (i.e., the phylogenetic flexible analysis; Motani & Schmitz, 2011; Schmitz & Motani, 2011). The best rate of habitat prediction occurred when both carnivorans (hit rate = 90.63%; TAU = 85.94%, Table 7) and ungulates (hit rate = 73.02%; TAU = 59.52%, Table 9) were analyzed separately suggesting that the fundamental shape of the humerus differ between Carnivora and ungulates. However, most of the misclassification cases were due to the species included in the mixed vegetation category, which is intermediate between open and closed and implies more generalized humerus morphologies. Applying the best phylogenetic FDA model to the whole sample, the misclassified rate was 29.47%. However, 92.85% (of 29.47%) misclassification cases concerned species living in open or closed environments which were reclassified as living in mixed vegetation type and *vice versa*. And this limitation may also have affected the fossil predictions.

Finally, we found that humerus morphologies gradually changed from species showing morphotypes facilitating maneuverability in closed vegetation to morphotypes facilitating running speed and long‐distance locomotion in open vegetation. Traditional shape variables were selected as the best predictors in paleoecological reconstructions and so this approach is also recommended for future studies aiming to explore the potential of other isolated long bones for paleovegetation cover reconstruction.

## AUTHOR CONTRIBUTIONS

**Carmela Serio:** Conceptualization; data curation; formal analysis; investigation; methodology; software; visualization; writing – original draft. **Richard P. Brown:** Conceptualization; methodology; supervision; validation; writing – review and editing. **Marcus Clauss:** Conceptualization; methodology; supervision; validation; writing – review and editing. **Carlo Meloro:** Conceptualization; data curation; formal analysis; investigation; methodology; supervision; validation; writing – review and editing; project administration.

## FUNDING INFORMATION

This research received support from the SYNTHESYS+ Project http://www.synthesys.info/ which is financed by the European Commission via the H2020 Research Infrastructure program.

## CONFLICT OF INTEREST STATEMENT

The authors declare no conflicts of interest.

## Supporting information


**Appendix A:** Supporting information.

**Appendix B:** Supporting information.

**Appendix C:** Supporting information.

**Appendix D:** Supporting information.

## Data Availability

Data are supplied as Appendix A.
